# Biological Aging Acceleration in Major Depressive Disorder: A Multi‐Omics Analysis

**DOI:** 10.1111/acel.70310

**Published:** 2025-12-04

**Authors:** Breno Satler Diniz, Shangshu Zhao, Gabin Drouard, Eero Vuoksimaa, Miina Ollikainen, Eric J. Lenze, Ming Xu, Richard H. Fortinsky, George A. Kuchel, Jaakko Kaprio, Chia‐Ling Kuo

**Affiliations:** ^1^ UConn Center on Aging University of Connecticut Health Center Farmington Connecticut USA; ^2^ Department of Psychiatry University of Connecticut Health Center Farmington Connecticut USA; ^3^ Institute for Molecular Medicine Finland, HiLIFE University of Helsinki Helsinki Finland; ^4^ Department of Psychiatry Washington University in St Louis St Louis Missouri USA; ^5^ Department of Biochemistry, Molecular Biology and Biophysics University of Minnesota St Paul Minnesota USA; ^6^ Department of Public Health Sciences University of Connecticut Health Center Farmington Connecticut USA; ^7^ The Cato T. Laurencin Institute for Regenerative Engineering University of Connecticut Health Center Farmington Connecticut USA

**Keywords:** biological aging, DNA methylation, major depression, Mendelian randomization, proteomics

## Abstract

Major depressive disorder (MDD) is linked to a higher risk of premature aging, but the mechanisms underlying this association remain unclear. Using data from two population cohorts (UK Biobank and Finnish Twin Cohort), we evaluate the relationship between systemic and organ‐specific proteomic and epigenetic aging acceleration and MDD. A lifetime history of MDD was associated with accelerated proteomic aging at both systemic and organ‐specific levels—including the brain—in both cohorts, with stronger associations than those observed with systemic epigenetic aging. Systemic and brain‐specific proteomic aging acceleration were linked to higher risks of incident MDD and a greater risk of Alzheimer's disease, related dementia, and mortality among individuals with MDD in the UK Biobank. Evidence of depressive episode remission attenuated the association between MDD and systemic and brain‐specific proteomic aging acceleration. Finally, Mendelian randomization analyses revealed a causal effect of MDD on systemic and brain‐specific proteomic aging acceleration. Our results suggest a strong bidirectional association between MDD and biological aging acceleration. Biological aging acceleration, assessed by proteomic systemic and organ‐specific clocks, can serve as a novel therapeutic target for treating MDD and for mitigating the long‐term risks of adverse health outcomes associated with this condition.

## Graphical abstract

A lifetime history of MDD was associated with accelerated proteomic aging at both systemic and organ‐specific levels—including the brain, based on observational and Mendelian Randomization analyses. Our results suggest a complex association between MDD and biological aging acceleration, which can be a novel therapeutic target for this condition.

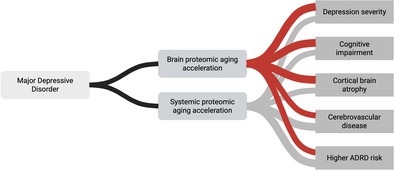

**Abbreviations**
ADRD: Alzheimer's disease and related dementia
ATC: anatomical therapeutic classification
BA: biological aging
BAA: biological aging acceleration
BAC: biological aging clocks
EH‐EPI: essential hypertension epigenetics study
GWAS: genome‐wide association studies
HPS: healthspan proteomic score
IDP: brain MRI imaging‐derived phenotypes
MDD: major depressive disorder
MR: Mendelian randomization
PAC: proteomic aging clock
PHQ: patient health questionnaire

## 1 Introduction

Major depressive disorder (MDD) is one of the most common mental disorders across the lifespan. Its prevalence varies in different populations, and the 12‐month and lifetime prevalence estimates in the US are 10.4% and 20.6%, respectively (Hasin et al. 2018). In addition to its high prevalence, it also ranks among the five most disabling disorders worldwide (Whiteford et al. 2013). Several factors contribute to the disability associated with MDD beyond the severity of psychopathology. For example, a lifetime history of MDD is associated with a higher risk of medical multimorbidity, including cardiovascular, cerebrovascular disease, and metabolic disorders, and decreased healthspan (Leung et al. 2012; Richmond‐Rakerd et al. 2021). MDD is also one of the most significant risk factors for mortality (including deaths by suicide), Alzheimer's disease and related dementia (ADRD), and frailty (Diniz et al. 2013; Soysal et al. 2017; Walker et al. 2015). Notably, these are features commonly associated with advancing chronological aging, suggesting that suffering from MDD may lead to premature aging.

Despite the evidence suggesting that MDD is associated with a premature aging phenotype, few studies have evaluated its association with biological aging clocks (BAC). In a community‐based study, individuals with MDD showed significantly accelerated epigenetic aging compared to never‐depressed individuals, and a significant dose‐effect with increasing symptom severity in the overall sample (Han et al. 2018). Another study, using a 2nd generation DNA methylation clock, the GrimAge, also showed that individuals with MDD presented with biological aging acceleration compared to non‐depressed controls (Protsenko et al. 2021). Another study, focusing on BAC derived from clinical chemistry measures (i.e., the Klemera‐Doubal method, Biological Aging and PhenoAge), showed that biological aging acceleration (BAA) was associated with a higher risk of MDD diagnosis (Gao, Geng, et al. 2023). However, another recent study did not find significant associations between MDD and BAA, as measured by different DNA methylation clocks (e.g., HorvathAge, HannumAge, SkinBloodAge, PhenoAge, and GrimAge) (Tanifuji et al. 2023). These studies have important limitations, including relatively small sample sizes, cross‐sectional study design, a focus on BAC derived only from DNA methylation or clinical chemistry measures, and the potential impact of antidepressant use on BAA.

To address the limitations of previous studies, we investigated the association between MDD and biological aging acceleration, focusing on recently developed systemic and organ‐specific BAC based on proteomic and DNA methylation data. We evaluated both cross‐sectional and longitudinal associations between MDD and systemic and organ‐specific BAA. Next, we examined the causal effects between MDD and biological aging acceleration using Mendelian randomization methods. Our primary hypotheses were that (1) individuals with a history of MDD show accelerated biological aging at systemic and organ‐specific (e.g., brain) levels, and that (2) systemic and organ‐specific BAA predicts the incidence of MDD. We also hypothesized a causal effect between MDD and BAA. Further, we explored if the use of antidepressant medication is associated with an attenuation of biological aging acceleration, and if accelerated systemic and organ‐specific biological aging is associated with adverse health outcomes among individuals with MDD, for example, higher risk of ADRD and mortality. Our primary analyses were conducted using data from the UK Biobank (UKB) cohort (Sudlow et al. 2015), and the findings were independently validated in the Essential Hypertension Epigenetics Study (EH‐Epi) study, a sub‐cohort of the Finnish Twin Cohort (FTC) (Kaprio et al. 2019).

## 2 Methods

### 2.1 UK Biobank

UKB is a large population‐based prospective study recruiting volunteers aged 40–69 years between 2006 and 2010 (Sudlow et al. 2015). For the current analysis, we included 53,014 participants who had proteomic data available for the calculation of systemic and organ‐specific proteomic aging measures (Sun et al. 2023). MDD diagnosis was ascertained using the first‐occurrence data released by the UKB, including multi‐source data based on ICD‐10 codes (Table S1). Patient Health Questionnaire‐4 (PHQ‐4) scores were derived as previously reported (Table S1) (X. Gao, Geng, et al. 2023). To identify possible cases of mild depression or those that were not readily diagnosed in the UK Biobank, we included individuals with a total PHQ‐4 score of ≥ 3 or a lifetime MDD diagnosis (broad MDD category). Additionally, we defined a current MDD episode by a PHQ‐4 score ≥ 3, while remitted MDD was defined as participants with a lifetime diagnosis of MDD and a current PHQ‐4 score < 3. Participants without a history of MDD were classified as never depressed.

Participants with a diagnosis of psychotic disorders (e.g., schizophrenia and bipolar disorder), or neurological disorders (Table S1) and those with any missing baseline covariate data were excluded, resulting in a final sample of 50,297.

### 2.2 Biological Aging Proteomic Measures

The proteomic aging clock (PAC) and healthspan proteomic score (HPS) are systemic proteomic biomarkers of biological aging (Kuo et al. 2024, 2025). They were developed using normalized protein expression (NPX) data from 2920 proteins measured in the UKB with the Olink Explore 3072 assay to predict mortality and healthspan. Since higher HPS values indicate better systemic biological health, we use its inverse values (1‐HPS) to facilitate the comparison with other proteomic and epigenetic aging clocks. Additionally, we included organ‐specific proteomic clocks for eight tissues: brain, adipose, immune system, heart, arteries, intestine, kidneys, and liver (Goeminne et al. 2024). Additional details related to the calculation of the proteomic aging clocks can be found in the Supporting Information.

### 2.3 Outcomes

We examined the association between a history of MDD and proteomic aging measures at baseline. We also examined the associations between PAC, HPS, and the brain‐specific proteomic aging clock with cognitive function and brain MRI image‐derived phenotypes (IDPs) (Table S1). Cognitive function was assessed through online cognitive tests, with measurement details available elsewhere (Fawns‐Ritchie and Deary 2020). Upon follow‐up, we evaluated the association between baseline proteomic aging measures and incident MDD, incident ADRD, and mortality, including deaths by suicide (Table S1).

### 2.4 Covariates

We selected a priori covariates for evidence of associations with MDD and BA acceleration, including age, sex, ethnicity, education, Townsend deprivation index, body mass index, smoking status, hypertension, and diabetes diagnosis status at baseline (Table S1). The use of antidepressants in participants with MDD was assessed using self‐reported medication data at baseline, linking UKB prescription codes to ATC codes of antidepressants (Table S2).

### 2.5 Statistical Methods

Linear regression models were used to examine the associations between MDD status and these proteomic aging measures at baseline, adjusting for chronological age and other covariates. Residuals from linear regression models for PAC, HPS, and the brain proteomic aging clock were correlated with cognitive function and brain MRI IDPs using Spearman correlation. Models were adjusted for chronological age and the time gap between protein measurements and the cognitive function measure or IDP.

Cox regression models were used to investigate the associations of baseline proteomic aging measures with ADRD and mortality during follow‐up since recruitment. In these survival analyses, participants were censored at the earliest of death or the last follow‐up date of hospital inpatient data for ADRD and were censored at the last follow‐up of hospital inpatient data for mortality. Models were adjusted for baseline variables. *p*‐values were adjusted for multiple testing using the Benjamini–Hochberg FDR method. Statistical analyses were performed using R version 4.4.3. Additional details about the statistical methods are available in the Supporting Information.

## 3 The Essential Hypertension Epigenetics (EH‐Epi) Study

### 3.1 Cohort Description

The external replication analyses were conducted in an independent sample of twins from the Finnish Twin Cohort (FTC), specifically those who participated in the Essential Hypertension Epigenetics (EH‐Epi) study (Kaprio et al. 2019). During in‐person visits between 2013 and 2015, the twins provided fasting blood samples, clinical and physiological measurements (Huang et al. 2020). Additional details about the EH‐Epi study are available in the Supporting Information.

### 3.2 Proteomic Data

Proteomic data were obtained using the Olink Explore 3072 platform (Olink Proteomics AB, Uppsala, Sweden) from plasma samples of 415 EH‐Epi twins (Drouard et al. 2024). After quality control, normalized protein expression (NPX) data were used to compute PAC, HPS, and organ‐specific proteomic aging clocks for 401 twins.

### 3.3 DNA Methylation Data

DNA methylation levels were measured using the Infinium Illumina HumanMethylation450K array and preprocessed with the R package ‘meffil’ (Min et al. 2018). We used six previously generated epigenetic age estimates, available for 379 of the 401 Finnish twins (Sehovic et al. 2023) (i.e., Horvath, Hannum, DNAm PhenoAge, GrimAge, GrimAge2, and DunedinPACE).

### 3.4 Depression Assessments

- Center for Epidemiological Study—Depression 20 items (CES‐D‐20) scale. A score of 20 or higher indicates a clinically relevant level of depression.
- Self‐reported physician diagnosis from the 2011 questionnaire conducted 1–2 years before blood sampling.
- Broad MDD classification is defined as either a self‐reported physician diagnosis or current use of antidepressant medication at the time of blood sampling.

### 3.5 Statistical Methods

We used Generalized estimating equation (GEE) models to examine the associations between depression measures (CES‐D total score, CES‐D‐defined MDD, and broad MDD category) and BAA, as GEE models enable correction for non‐independence of observations induced by family relatedness. Models were adjusted for chronological age and sex. For each BA measure, *p*‐values were adjusted for multiple testing using the Benjamini–Hochberg FDR method, with significance assessed at the 5% level.

### 3.6 Mendelian Randomization Analysis

Observational studies are susceptible to potential biases, including unmeasured confounding and reverse causation, which can undermine causal inference. To address this issue, we conducted a bidirectional Mendelian randomization (MR) analysis to assess the causal relationship between MDD and accelerated proteomic aging. To evaluate the causal effect of proteomic aging on MDD, we selected genetic instruments associated with accelerated proteomic aging from genome‐wide association studies (GWAS) on PAC, HPS, and the brain proteomic aging clock (Table S3). For the causal effect of MDD on proteomic aging, genetic instruments for MDD (Table S4) were selected based on a recent meta‐GWAS (Major Depressive Disorder Working Group of the Psychiatric Genomics Consortium et al. 2025).

Two‐sample Mendelian randomization (MR) analysis was conducted using the inverse variance weighting (IVW) method (Burgess et al. 2013) as the primary approach for causal inference. To assess the robustness of our findings, we performed sensitivity analyses using additional MR methods: MR‐Egger regression, the Robust Adjusted Profile Score (MR‐RAPS) method, and MR‐PRESSO. All MR analyses were conducted using the R package *MendelianRandomization* v0.10.0. Additional details about the Mendelian randomization methods are available in the Supporting Information.

## 4 Results

### 4.1 Cross‐Sectional Analysis

A total of 50,297 participants with proteomic data available in the UKB were included in these analyses. Participants had a mean age of 57 years (range, 39–70), with the majority being female (54%) and of European ancestry (94%). Approximately 33% of participants held a college or university degree. The prevalence of a lifetime history of MDD was 8.9%. Table S5 provides additional descriptive characterization of the sample included in the analysis.

At baseline, participants with a history of MDD (*n* = 4477) were younger, more likely to be female, and socioeconomically disadvantaged compared to those without MDD (*n* = 45,820). They also had a higher prevalence of chronic diseases, such as diabetes and hypertension. Additionally, individuals with a history of MDD were more likely to have higher PHQ‐4 scores at the time of recruitment compared to those without a history of MDD. Approximately 51% of participants with a history of MDD were using antidepressants at the time of recruitment (Table S6).

**FIGURE 1:**
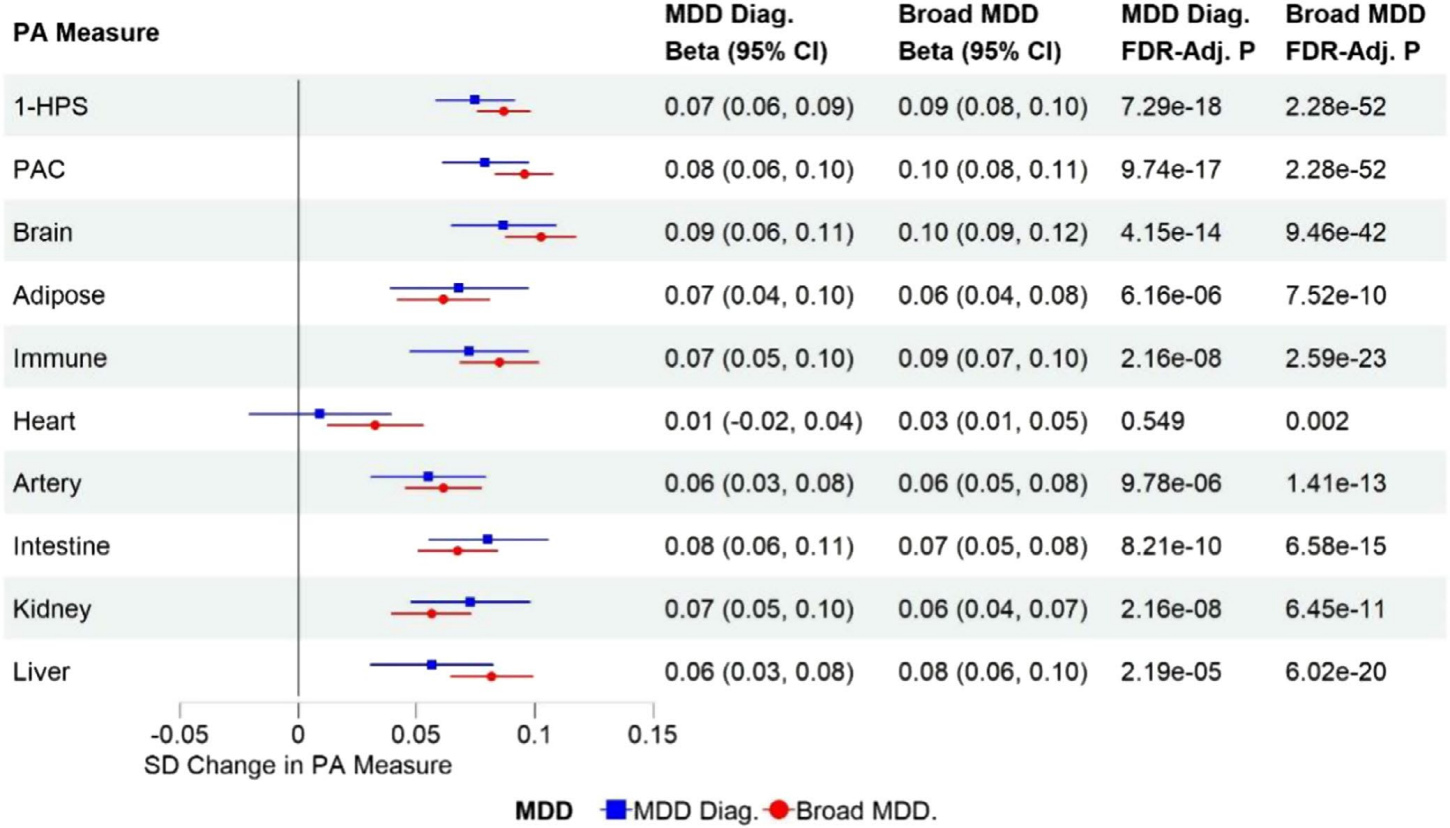
Associations between proteomic aging (PA) measures (*z*‐scores) and a history of MDD at baseline after adjusting for covariates (MDD Diag., diagnosed with MDD before or at baseline; Broad MDD, diagnosed with MDD before or at baseline or PHQ‐4 positive at baseline) MDD, major depressive disorder; MDD diag., based on ICD‐10 codes (Table S1 for details); Broad MDD, Total PHQ‐4 score of ≥ 3 or lifetime MDD diagnosis.

**FIGURE 2:**
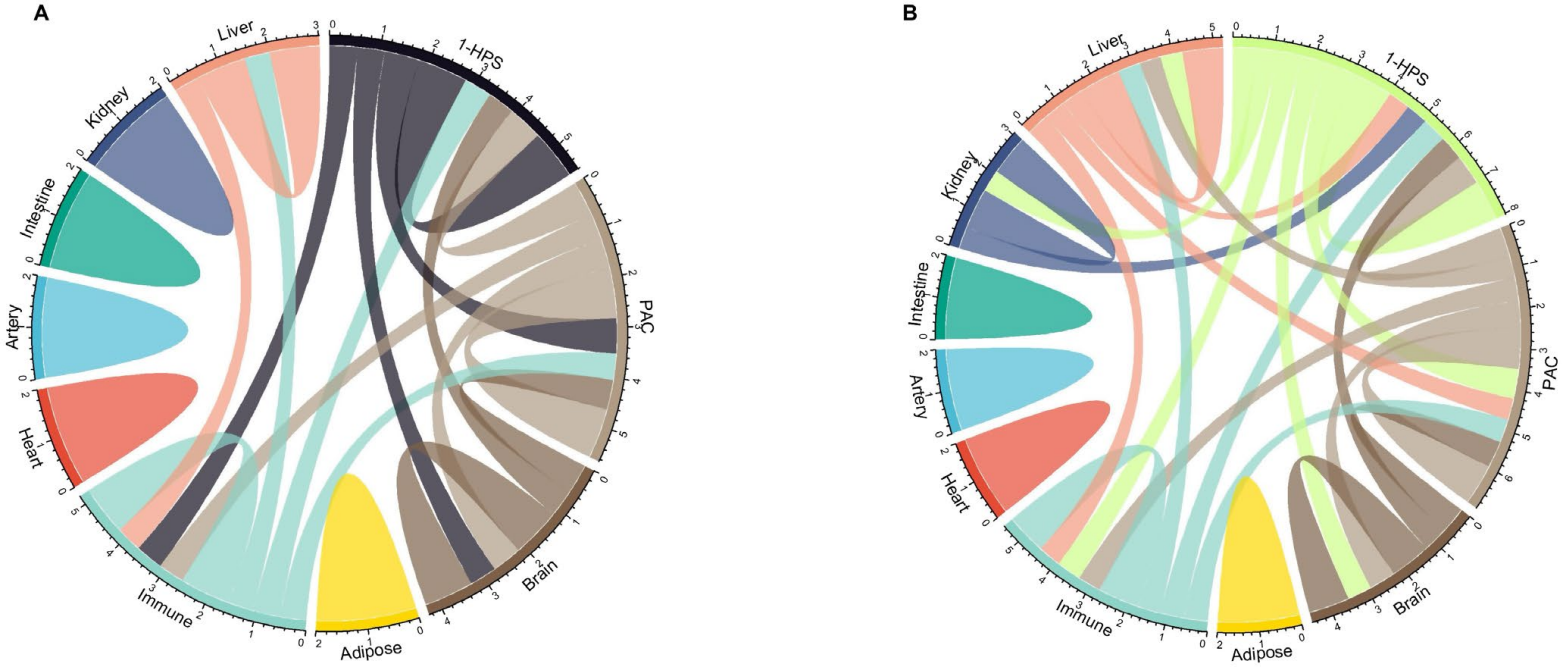
Interaction network across systemic and organ‐specific proteomic aging in individuals without MDD (A) and with MDD (B) at baseline. Interaction network showing Pearson *r* correlations ≥ 0.5, indicating moderate‐to‐strong associations.

The history of MDD was significantly associated with lower HPS, higher PAC, and higher organ‐specific proteomic aging measures after adjusting for chronological age and other covariates, suggesting systemic and organ‐specific biological aging acceleration in MDD (Figure 1). The associations were more robust in the broad MDD category (ICD‐10 diagnosis or PHQ‐4 > 3), with the brain proteomic aging clock showing the strongest association (Figure 1). Next, we evaluated the associations between systemic and organ‐specific proteomic aging clocks in individuals with MDD and those without depression. We found that the association among the proteomic aging clocks was stronger within the MDD groups than in those without depression, suggesting that mechanisms related to MDD are driving the acceleration of biological aging in this sample (Figure 2 and S1).

Additional analyses revealed that individuals with MDD in remission showed significantly higher HPS and PAC levels compared to individuals without a lifetime diagnosis of MDD. They also showed significantly elevated levels of multiple organ‐specific biological aging clocks, most significantly in the brain, adipose, and intestine tissues, indicating that even in remission, individuals with a lifetime history of MDD may experience persistent biological aging acceleration in multiple organs that is not fully resolved even after the resolution of the depressive episode (Figure S2). Individuals with MDD who were prescribed antidepressants also presented with significantly higher systemic and organ‐specific BAA (including brain proteomic aging clock) compared to those not prescribed antidepressants (Figure S3a). Such associations were consistent regardless of individuals with MDD being acutely depressed or in remission. Next, we analyzed the impact of individual classes of antidepressants (tricyclics (TCA), selective serotonin reuptake inhibitors (SSRI), serotonin and noradrenaline reuptake inhibitors (SNRI), and other antidepressants) on BAA. In general, all antidepressant classes were associated with higher systemic BAA (Figure S3b). Interestingly, SSRIs and SNRIs, the most commonly prescribed antidepressant classes, were significantly associated with higher proteomic brain BAA.

Systemic and brain proteomic aging acceleration were correlated with worse cognitive performance, particularly executive dysfunction, whole‐brain and regional cortical atrophy in brain areas critical for cognitive and emotional processing, and a higher cerebrovascular burden, as measured by white matter hyperintensities, after adjusting for chronological age and the time gap between blood collection and cognitive assessment or brain MRI scan (Figures S4 and S5; Table S7).

Since age and sex can significantly influence the effects of MDD and BAA (Kuo et al. 2025; Navrange et al. 2025), we conducted a sensitivity analysis based on sex (male vs. female) and age groups (40–59 years vs. 60 years and older). In general, the effects of MDD on systemic and organ‐specific BAA were significant in both age groups and sexes. We did not find a significant interaction between sex and MDD diagnosis on systemic and brain‐specific proteomic aging clocks (Figure S6). On the other hand, we found a significant interaction between age group and MDD diagnosis, indicating that younger adults with MDD had a more intense BAA in the systemic and brain‐specific proteomic aging clock compared to older adults. These results suggest that younger adults may be more susceptible to the accelerated aging effect of MDD than older adults, and possibly, more vulnerable to the associated adverse health outcomes (Kuo et al. 2025).

### 4.2 Longitudinal Analyses

**FIGURE 3:**
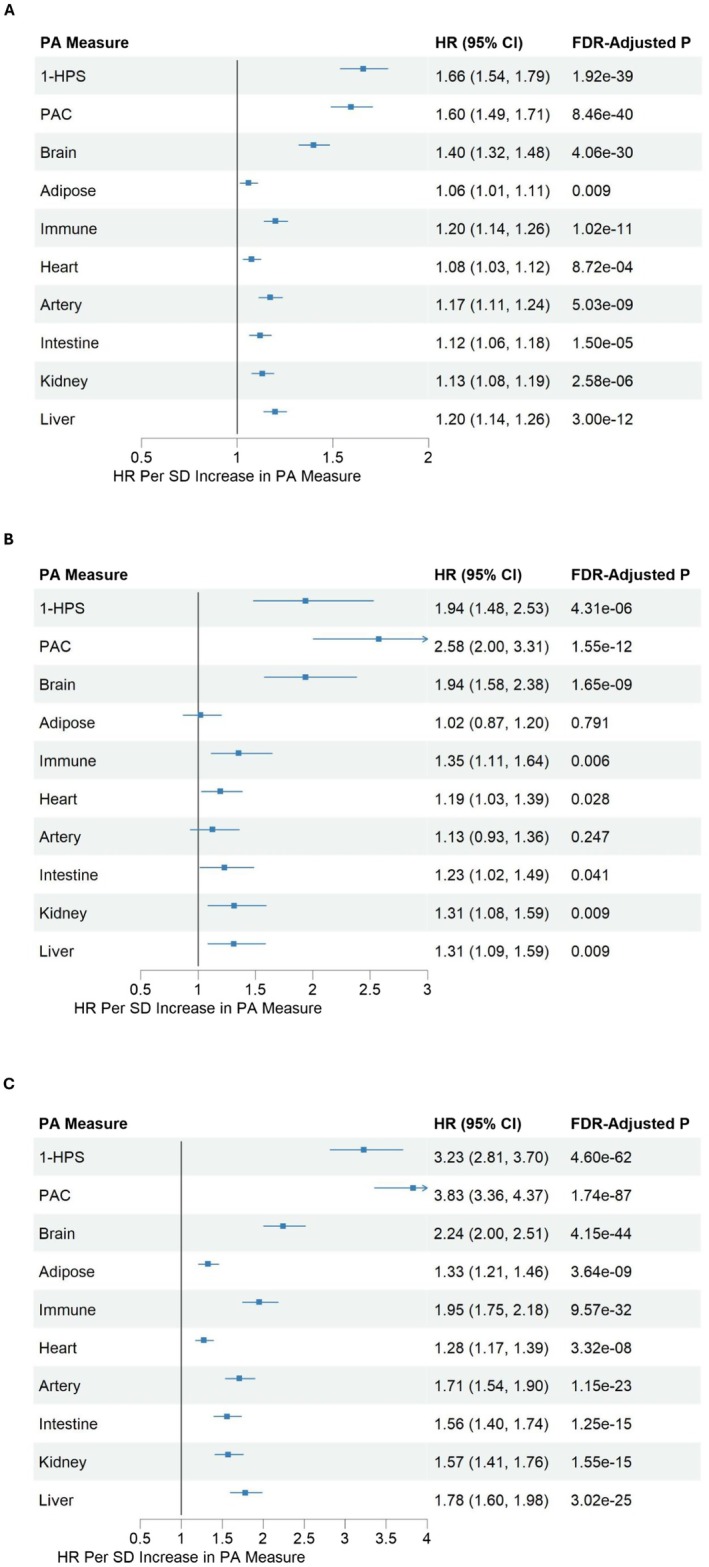
Associations between proteomic aging (PA) measures (*z*‐scores) at baseline and incident MDD and adverse health outcomes during follow‐up after adjusting for covariates. (A) Incident MDD. (B) Incident ADRD in individuals with MDD at baseline. (C) Mortality.

Next, we evaluated whether accelerated biological aging predicts the incidence of MDD. Among 45,820 participants without a lifetime diagnosis of MDD at baseline, 2280 were diagnosed with MDD over a mean follow‐up of 13.3 years, with a mean age at diagnosis of 63.9 years (SD = 9.6). Our analyses revealed that a lower HPS was associated with a higher risk of MDD upon follow‐up (a 66% higher risk per 1 SD lower HPS) (Figure 3). Higher PAC was associated with an increased risk of incident MDD (a 60% higher risk per 1 SD increase in PAC) (Figure 3). Additionally, the proteomic brain BAA also increased the risk of incident MDD (a 40% higher risk per 1 SD increase in brain proteomic aging clock) (Figure 3).

During the mean follow‐up of 13.3 years, the incidence of ADRD and the mortality rate in individuals with MDD (*n* = 4477) were 3.9% and 11.8%, respectively. Higher systemic and proteomic brain BAA predicted the risk of ADRD and mortality in these individuals. More specifically, we found that a lower HPS increased the risk of ADRD and all‐cause mortality by 94% and 223%, respectively (Figure 3), while one SD increase in PAC elevated the risk of ADRD and all‐cause mortality by 158% and 223%, respectively, and one SD increase in brain proteomic BAA elevated the risk of ADRD and all‐cause mortality by 94% and 124%, respectively (Figure 3).

### 4.3 Differential Impact of MDD on Proteomic Versus Epigenetic Biological Aging Acceleration

Biological aging clocks have been trained using different molecular types, which may convey distinct biological information. For example, DNA methylation can be viewed as a molecular memory in cells in response to environmental influences, is relatively stable over time and transmitted with high fidelity during DNA replication (Kim and Costello 2017). On the other hand, proteins are more dynamic and proximal indicators of physiological or pathological states (Moaddel et al. 2021). Using data from the EH‐Epi study, we evaluated the association between depression and BAA, based on proteomic and epigenetic clocks.

The EH‐Epi study included 401 twin individuals with available proteomic data, among whom 379 also had DNA methylation data for epigenetic age estimation. Among the 401 participants, 41% were female, and 47% had never smoked. The mean age was 62.3 years (range: 56–70), with a mean BMI of 27.3 (SD = 4.9; range: 18–46). Regarding depression, the mean CES‐D score was 10.1 (range: 0–47). Based on different depression definitions, 44 (11%) participants met the criteria for CES‐D‐defined MDD, and 63 (16%) reported a physician‐diagnosed MDD. Broad MDD, defined as self‐reported physician‐diagnosed MDD (*n* = 63) and/or antidepressant use (*n* = 29), was identified in 75 participants (19%). Correlations between proteomic and epigenetic clocks, adjusted for chronological age, are presented in Figures S7 and S8.

**FIGURE 4:**
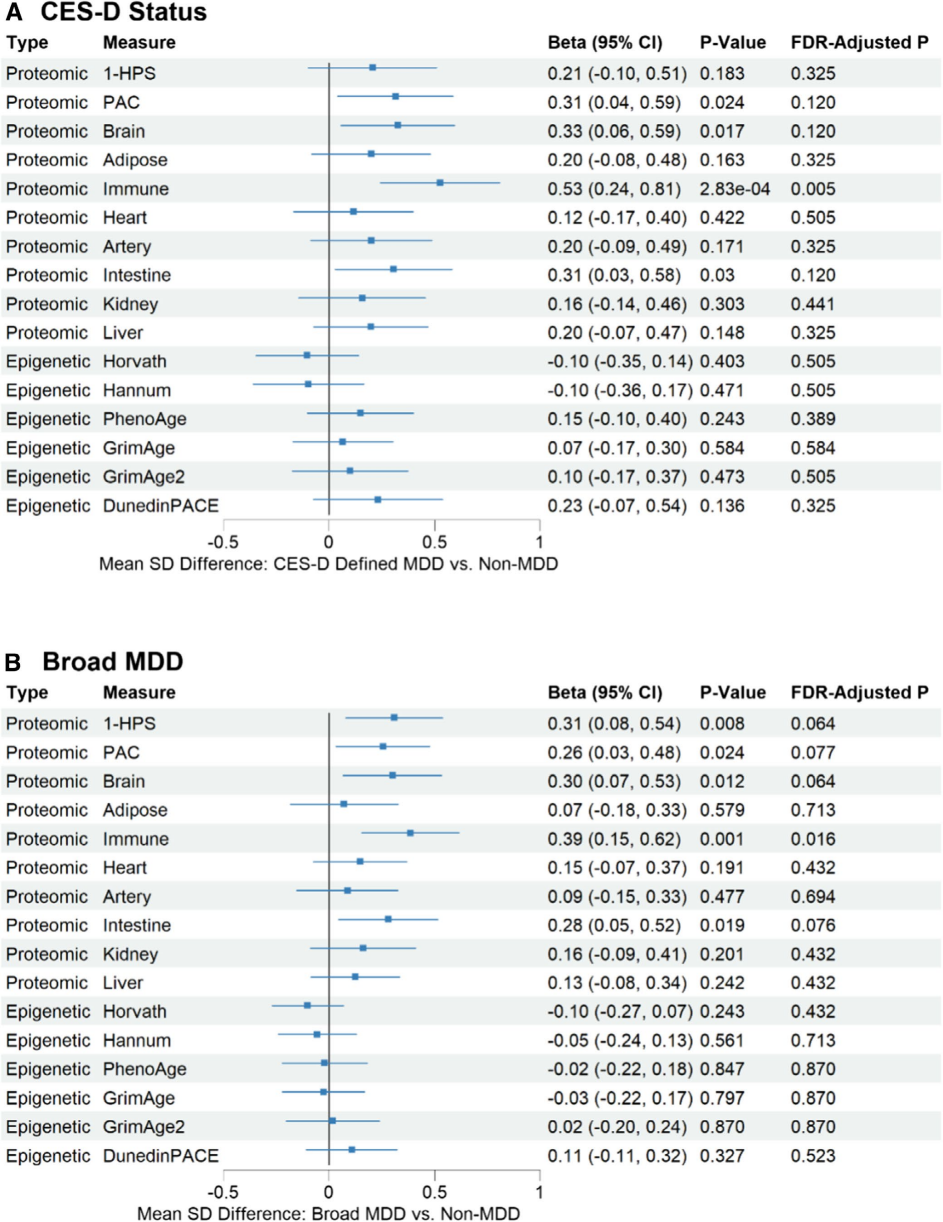
Associations of CES‐D status (CES‐D score ≥ 20 vs. < 20) and broad MDD (self‐reported physician diagnosis or antidepressant use vs. others) with proteomic or epigenetic aging clocks after inverse normal transformation, adjusted for chronological age and other covariates. Broad MDD, total PHQ‐4 score of ≥ 3 or lifetime MDD diagnosis; CES‐D, Center for Epidemiologic Studies Depression Scale; MDD, major depressive disorder.

Individuals with CES‐D ≥ 20 showed evidence of biological aging acceleration based on proteomic aging measures (i.e., higher PAC, brain, immune and intestine‐specific biological aging acceleration) (Figure 3). However, CES‐D status showed minimal association with epigenetic aging across all measures (Figure 4). A sensitivity analysis using a broader MDD definition—based on self‐reported physician diagnosis and/or current antidepressant use—yielded similar results (Figure 4). These analyses independently replicate the association between depression and systemic as well as organ‐specific proteomic biological aging acceleration, particularly in the brain. Moreover, they highlight the differential impact of depression on biological aging measures, with effects being more pronounced in proteomic aging clocks than in epigenetic‐based measures.

### 4.4 Mendelian Randomization Analysis

Observational studies are subject to potential biases, such as unmeasured confounding and reverse causation, which can undermine causal inference (Smith and Ebrahim 2002). To address this issue, we conducted a bidirectional Mendelian randomization analysis to assess the causal relationship between MDD and accelerated proteomic aging.

**FIGURE 5:**
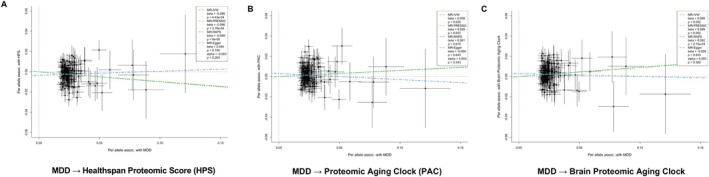
Mendelian randomization analysis for the effects of MDD on PAC, HPS, and brain proteomic aging clock. →, arrow indicates the direction of the association; MDD, major depressive disorder.

We first evaluated the causal effects of MDD on biological aging acceleration. Our findings indicate that genetically determined susceptibility to MDD is causally associated with systemic and brain proteomic aging acceleration (HPS: IWV standardized β (mean SD change in HPS per one unit increase in genetically determined log odds for MDD) = −0.096, *p* < 0.001; PAC: IVW standardized *β* = 0.059, *p* = 0.035; and Brain: IVW standardized *β* = 0.089, *p* = 0.002) (Figure 5). The results were consistent across different MR methods (Table S8). These analyses revealed that MDD has the strongest causal effect on HPS and proteomic brain aging, reinforcing its association with biological aging acceleration at systemic and brain levels. However, we found no significant causal effect of genetically determined proteomic aging on MDD (Figure S9).

## 5 Discussion

Several mechanisms have been proposed as potential links to the development of premature aging phenotypes in MDD (Lorenzo et al. 2023). In the current study, systemic and organ‐specific (in particular, in the brain) proteomic aging acceleration was significantly greater in individuals with a history of MDD, particularly among those showing evidence of an acute depressive episode. Importantly, these findings were replicated in an independent cohort, the EH‐Epi study. Systemic and brain proteomic aging acceleration was associated with an increased risk of incident MDD upon follow‐up, poorer cognitive performance, global and regional cortical brain atrophy, a higher burden of cerebrovascular disease, and a higher risk of adverse health outcomes (e.g., incidence of ADRD and mortality). Lastly, Mendelian randomization analysis suggested a causal relationship between MDD, systemic, and brain proteomic aging acceleration. Overall, our study provides robust evidence of a complex link between MDD and accelerated biological aging and suggests a potential causal role for MDD in systemic and brain BAA in middle‐aged and older adults.

Prior studies have shown evidence of modestly accelerated brain aging in MDD based on neuroimaging data. However, the prognostic relevance of structural brain aging is limited. Most studies presented a small effect size (Seitz‐Holland et al. 2024), suggesting that MDD may have a small impact on brain aging, at least on the structural level. In contrast, our findings demonstrate a strong association between proteomic brain aging acceleration and MDD at baseline and follow‐up. Interestingly, we found a significant association between proteomic brain BAA and regional brain atrophy, cerebrovascular disease, and poorer cognitive performance. Moreover, proteomic brain BAA has major prognostic relevance, since it is associated with persistent depressive episodes even under antidepressant treatment, and an increasing risk of adverse health outcomes, such as ADRD and mortality. These findings have important implications. First, they suggest that the effect of MDD on brain aging is more robust at the molecular than at the structural level. Second, proteomic brain BAA might be an underlying mechanism of deteriorating brain health, worse long‐term health outcomes, and an indicator of treatment resistance in MDD. It is worth noting that these findings were based on cross‐sectional data and in a much smaller sample in the UKB due to the limited overlap of individuals with available neuroimaging and proteomic data. Therefore, these findings need to be replicated in future studies with a longitudinal design, standardized antidepressant treatment, and evaluation of the co‐trajectory of proteomic biological aging, depressive symptoms, and brain health parameters to clarify the impact of MDD on proteomic brain aging.

The history of MDD is a well‐established risk factor for ADRD (Diniz et al. 2013). Recent evidence does not support that MDD is associated with the build‐up or acceleration of amyloid‐β or tau accumulation in the brain, the primary pathological hallmarks of ADRD (Casteele et al. 2024; Wiels et al. 2025); but suggests alternative mechanisms for this robust epidemiological association, many of them linked to different hallmarks of biological aging (Diniz et al. 2025). Our finding that brain proteomic aging acceleration was strongly associated with a higher risk of ADRD upon follow‐up can provide an alternative mechanistic explanation for such associations. From this perspective, proteomic brain aging acceleration may decrease resilience (i.e., brain reserve) against neurotoxic insults (i.e., amyloid‐β deposition), thereby reducing the threshold for the manifestation of cognitive impairment and the development of dementia. Additionally, proteomic brain aging acceleration can interact with other pathological processes (e.g., cerebrovascular disease, neuroinflammation), culminating in an elevated risk of ADRD in MDD.

Our analysis revealed that individuals with remitted MDD exhibited persistent systemic and brain proteomic aging acceleration compared to never‐depressed individuals, although this association was weaker than in those with an acute depressive episode. These observations may suggest that while the successful treatment of a depressive episode may attenuate its harmful effects on biological aging, it may not fully reverse them. Conversely, we found that the current use of antidepressants was associated with systemic and brain proteomic aging acceleration in both remitted and acute depressive episodes. The same pattern was evident when we analyzed specific antidepressant classes (TCA, SSRI, SNRI). These contradictory findings may stem from a common bias in observational studies where antidepressant use often indicates more severe depressive episodes (i.e., confounding by indication), making it difficult to distinguish the effects of medication from the severity of disease (Andrade 2022; Haro et al. 2018). These results can also provide a mechanistic explanation for previous findings showing a marginal benefit of successful antidepressant treatment in reducing mortality and the risk of dementia in MDD (Gallo et al. 2013; Subramaniapillai et al. 2021), as well as the notion that individuals with more severe depressive symptoms may be at the highest risk of developing dementia (Kaup et al. 2016). However, we cannot exclude the fact that the use of antidepressants, despite improving symptoms of depression, may trigger and perpetuate processes contributing to biological aging acceleration in MDD. Future studies with a longitudinal design and careful control of antidepressant use are necessary to disambiguate these associations.

A large body of literature suggests that peripheral systems, like the immune system, cardiovascular system, adipose tissue, and the gut‐brain axis, exert significant influence on MDD pathophysiology and long‐term outcomes (Drevets et al. 2022; Gao, Wang, et al. 2023; Khan et al. 2021). Importantly, these organs are critical for maintaining brain health and function by regulating a proper homeostatic environment, delivering energy substrates for metabolism, regulating immune function, and clearing waste (Smith et al. 2024). Our results provide robust evidence that biological aging acceleration in multiple organs has a bidirectional association with MDD, particularly in the immune system, liver, arteries, kidneys, and intestines. These findings reinforce the robust evidence implicating vascular dysfunction and inflammaging in the development of MDD (Aizenstein et al. 2016; Lorenzo et al. 2023). They also corroborate the perspective that MDD affects multiple peripheral organs, not just the brain, and that dysfunction in peripheral organs can increase vulnerability to MDD development. Interestingly, accelerated biological aging of adipose tissue was not strongly associated with baseline or incident MDD, compared to other organ‐specific BAC, despite robust epidemiological evidence for the association between MDD and obesity (Penninx and Lange 2018), and the fact that obesity is one of the primary drivers of cellular senescence, a hallmark of biological aging, in this population (Diniz et al. 2019; Seitz‐Holland et al. 2023). A possible explanation is the set of markers used to build the adipose proteomic clock and how they might be associated with MDD (Goeminne et al. 2024). Given the strong association between obesity and MDD, future studies are necessary to clarify these associations.

Multiple biological aging clocks have been developed and validated using different omics modalities (e.g., DNA methylation, proteomics, metabolomics), and they may reflect distinct facets of biological aging (Ferrucci et al. 2018). In the independent replication cohort, we compared the magnitude of associations between MDD and BAC based on DNA methylation and proteomic data. Our results showed that associations between MDD and biological aging were primarily observed in proteomic clocks, including the brain proteomic aging clock. These findings have important implications. First, our results may reflect differences between the lifelong impact of MDD on biological aging processes, as captured by epigenetic changes, versus more proximal and dynamic effects, as reflected in proteomic alterations (Moaddel et al. 2021). These findings highlight the complexity and heterogeneity of biological aging processes related to MDD, and emphasize the importance of incorporating a multi‐omics approach to understand these complex relationships. Second, our findings highlight the importance of integrating multiple biological aging measures in observational studies and clinical trials evaluating the effects of geroscience‐guided intervention, since null findings may be due to the choice of surrogate measures of biological aging, rather than a true effect of a condition of interest or an intervention. Further studies are needed to confirm these preliminary observations and to disentangle how biological aging clocks, whether trained on DNA methylation or proteomic data, differ in MDD.

Using findings from the most recent and largest MDD GWAS analysis (Major Depressive Disorder Working Group of the Psychiatric Genomics Consortium et al. 2025), we demonstrated that genetically determined MDD is causally linked with both systemic and brain proteomic aging acceleration. Taken together, our findings provide evidence that MDD is not only a risk factor but also a primary etiological mechanism of biological aging acceleration in middle‐aged and older adults. Moreover, our findings provide a putative mechanism for the evidence that improvement of major depressive episodes can slow cognitive decline, lower the risk of developing ADRD, and reduce mortality among individuals with MDD (Gallo et al. 2013; Yang et al. 2023). These findings reinforce the importance of prevention, early recognition, and aggressive treatment of MDD (e.g., achieving sustained remission), as it can have a major impact on the trajectories of biological aging and mitigate the disability and long‐term adverse outcomes strongly associated with this condition across the lifespan. Moreover, they provide a mechanistic explanation for the clinical and epidemiological observations that health behaviors or medical conditions associated with accelerated biological aging (e.g., obesity, smoking, sedentarism, cardiometabolic disorders) (Nguyen and Corvera 2024; Thomas et al. 2023) are associated with a higher risk of MDD and that MDD increases the risk of multiple adverse health outcomes across the lifespan.

Our results should be interpreted considering the study's limitations. Despite the strong validity of EHR for the identification of MDD cases in the UK Biobank (Glanville et al. 2021), the lack of formal psychiatric interviews for the majority of UK Biobank participants does not allow for a fine‐grained characterization of the major depressive episode, such as currently depressed or in remission, age of onset, chronicity, the number of prior episodes, and trajectories of depressive symptoms after the diagnosis that might influence biological aging trajectories. Acute and remitted MDD were defined by the lifetime history of MDD and current PHQ‐4 scores. These are crude definitions of acute and remitted depression, and we could not evaluate factors that might also influence biological aging, such as the length of remission status and the amount of prior antidepressant exposure. Future studies are necessary to address the fine‐grained relationship between MDD and biological aging acceleration across the lifespan.

In conclusion, we provide a comprehensive analysis of the association between biological aging acceleration and MDD. Our findings support complex links between biological aging acceleration, including brain proteomic aging. We also found a robust causal effect of MDD on systemic and brain proteomic aging acceleration. Future studies should address whether interventions targeting biological aging can help prevent and treat MDD, reduce disability, and improve or extend healthspan in individuals with MDD.

## Author Contributions

Hypothesis generation, study design, interpretation of results, intellectual input, manuscript draft and final revision of the manuscript: Breno Satler Diniz. Study design, data analysis, interpretation of the results, intellectual input, and revision of the final manuscript draft: Shangshu Zhao, Gabin Drouard, Eero Vuoksimaa, Miina Ollikaine, Jaakko Kaprio. Interpretation of the results, intellectual input, and revision of the final manuscript draft: Eric J. Lenze, Ming Xu, Richard H. Fortinsky, George A. Kuchel. Hypothesis generation, study design, data analyses, interpretation of results, intellectual input, and final revision of the manuscript: Chia‐Ling Kuo.

## Funding

C.L.‐K., B.S.D., R.H.F., and G.A.K. are partially supported by the Claude D. Pepper Older American Independence Centers (OAIC) program: P30AG067988. G.D. has been supported by the doctoral programs of the University of Helsinki. Data collection in the twin cohort has been supported by the Academy of Finland (Grants 100499, 205585, 118555, 141054, 264146, 308248 to J.K., and 307339 and 328685 to M.O.) and the Academy of Finland Center of Excellence in Complex Disease Genetics (Grant 352792 to J.K.).

## Conflicts of Interest

The authors declare no conflicts of interest.

## Supporting information


**Table S1:** acel70310‐sup‐0001‐TableS1.pdf.

**Table S2:** acel70310‐sup‐0002‐TableS2.pdf.

**Table S3:** acel70310‐sup‐0003‐TableS3.pdf.

**Table S4:** acel70310‐sup‐0004‐TableS4.pdf.

**Table S5:** acel70310‐sup‐0005‐TableS5.pdf.

**Table S6:** acel70310‐sup‐0006‐TableS6.pdf.

**Table S7:** acel70310‐sup‐0007‐TableS7.pdf.

**Table S8:** acel70310‐sup‐0008‐TableS8.pdf.

**Data S1:** acel70310‐sup‐0009‐SupplementaryMethods.docx.

**Figure S1:** Spearman correlations between chronological age and proteomic aging clocks: (a) chronological age and proteomic aging clocks; (b) residuals of proteomic aging clocks after adjusting for chronological age in linear regression models.
**Figure S2:** Associations between proteomic aging (PA) measures (*z*‐scores) and a history of MDD at baseline after adjusting for covariates (Active: diagnosed with MDD and PHQ‐4 positive; Remitted: diagnosed with MDD and PHQ‐4 negative; Never Dep: never diagnosed with MDD).
**Figure S3:** Associations between antidepressant use (users vs. non‐users) and proteomic aging (PA) measures (*z*‐scores) in participants with MDD at baseline after adjusting for covariates (MDD Diag.: diagnosed with MDD before or at baseline; Active: diagnosed and PHQ‐4 positive; Remitted: diagnosed and PHQ‐4 negative).
**Figure S4:** Spearman correlations between the residuals of HPS, PAC, the brain proteomic aging clock, and cognitive function measures. Significance levels: *p* < 0.05 (*), *p* < 0.01 (**), *p* < 0.001 (***).
**Figure S5:** Spearman correlations between the residuals of HPS, PAC, the brain proteomic aging clock, and brain MRI image‐derived phenotypes (IDPs). IDPs are labeled if the FDR‐adjusted *p*‐value < 0.05 and the absolute Spearman correlation > 0.07, selected to ensure meaningful effect sizes while maintaining clarity.
**Figure S6:** Biological aging acceleration in MDD based on age group (40–59 years vs. 60 or more years) and sex (male vs. female).
**Figure S7:** Spearman correlations between chronological age, proteomic and epigenetic aging clocks
**Figure S8:** Spearman correlations between proteomic and epigenetic aging clocks after adjusting for chronological age in linear regression models.
**Figure S9:** Mendelian randomization analysis for the effects of PAC, HPS, and brain proteomic aging clock on MDD.

## Data Availability

Data access to the UK Biobank is granted upon application. The EH‐Epi study data used in the analysis is available through the Biobank of the Finnish Institute for Health and Welfare (https://thl.fi/en/web/thl‐biobank/forresearchers). It is available to researchers after a written application and following relevant Finnish legislation. The R code for computing PAC, HPS, and organ‐specific proteomic clocks and the GWAS summary statistics for proteomic aging acceleration based on PAC, HPS, and the brain proteomic aging clock can be obtained from the GitHub repository at https://github.com/kuo‐lab‐uchc/GWAS.
