# Supplementary material for: Biological Aging Acceleration in Major Depressive Disorder: A Multi‐Omics Analysis
Source: Aging Cell. 2025 Dec 4;25(1):e70310. doi: 10.1111/acel.70310 (PMC12741235; doi:10.1111/acel.70310)
Supplement: Supplementary file 1 — Table S1: acel70310‐sup‐0001‐TableS1.pdf. [file ACEL-25-e70310-s002.pdf]

Table S1. A list of UK Biobank field IDs to extract data for conditions in the healthspan definition, other medical

Instance 0 : Initial assessment visit (2006-2010) at which participants were recruited and consent given

Instance 2 : Imaging visit (2014+)

Variable	Instance	ICD codes	Field ID	Description
**Exclusion criteria**				
Non-MDD psychiatric or neurological disorders	-	F00	130836	Date F00 first reported (dementia in alzheimer's disease)
Non-MDD psychiatric or neurological disorders	-	F01	130838	Date F01 first reported (vascular dementia)
Non-MDD psychiatric or neurological disorders	-	F02	130840	Date F02 first reported (dementia in other diseases classified elsewhere)
Non-MDD psychiatric or neurological disorders	-	F03	130842	Date F03 first reported (unspecified dementia)
Non-MDD psychiatric or neurological disorders	-	F04	130844	Date F04 first reported (organic amnesic syndrome, not induced by alcohol and other psychoactive substances)
Non-MDD psychiatric or neurological disorders	-	F05	130846	Date F05 first reported (delirium, not induced by alcohol and other psychoactive substances)
Non-MDD psychiatric or neurological disorders	-	F06	130848	Date F06 first reported (other mental disorders due to brain damage and dysfunction and to physical disease)
Non-MDD psychiatric or neurological disorders	-	F07	130850	Date F07 first reported (personality and behavioural disorders due to brain disease, damage and dysfunction)
Non-MDD psychiatric or neurological disorders	-	F09	130852	Date F09 first reported (unspecified organic or symptomatic mental disorder)
Non-MDD psychiatric or neurological disorders	-	F60	130932	Date F60 first reported (specific personality disorders)
Non-MDD psychiatric or neurological disorders	-	F61	130934	Date F61 first reported (mixed and other personality disorders)
Non-MDD psychiatric or neurological disorders	-	F62	130936	Date F62 first reported (enduring personality changes, not attributable to brain damage and disease)
Non-MDD psychiatric or neurological disorders	-	F63	130938	Date F63 first reported (habit and impulse disorders)
Non-MDD psychiatric or neurological disorders	-	F64	130940	Date F64 first reported (gender identity disorders)
Non-MDD psychiatric or neurological disorders	-	F65	130942	Date F65 first reported (disorders of sexual preference)
Non-MDD psychiatric or neurological disorders	-	F66	130944	Date F66 first reported (psychological and behavioural disorders associated with sexual development and orientation)
Non-MDD psychiatric or neurological disorders	-	F68	130946	Date F68 first reported (other disorders of adult personality and behaviour)
Non-MDD psychiatric or neurological disorders	-	F69	130948	Date F69 first reported (unspecified disorder of adult personality and behaviour)
Non-MDD psychiatric or neurological disorders	-	F70	130950	Date F70 first reported (mild mental retardation)
Non-MDD psychiatric or neurological disorders	-	F71	130952	Date F71 first reported (moderate mental retardation)
Non-MDD psychiatric or neurological disorders	-	F72	130954	Date F72 first reported (severe mental retardation)
Non-MDD psychiatric or neurological disorders	-	F73	130956	Date F73 first reported (profound mental retardation)
Non-MDD psychiatric or neurological disorders	-	F78	130958	Date F78 first reported (other mental retardation)
Non-MDD psychiatric or neurological disorders	-	F79	130960	Date F79 first reported (unspecified mental retardation)
Non-MDD psychiatric or neurological disorders	-	F80	130962	Date F80 first reported (specific developmental disorders of speech and language)
Non-MDD psychiatric or neurological disorders	-	F81	130964	Date F81 first reported (specific developmental disorders of scholastic skills)
Non-MDD psychiatric or neurological disorders	-	F82	130966	Date F82 first reported (specific developmental disorder of motor function)
Non-MDD psychiatric or neurological disorders	-	F83	130968	Date F83 first reported (mixed specific developmental disorders)
Non-MDD psychiatric or neurological disorders	-	F84	130970	Date F84 first reported (pervasive developmental disorders)
Non-MDD psychiatric or neurological disorders	-	F88	130972	Date F88 first reported (other disorders of psychological development)
Non-MDD psychiatric or neurological disorders	-	F89	130974	Date F89 first reported (unspecified disorder of psychological development)
Non-MDD psychiatric or neurological disorders	-	F99	130990	Date F99 first reported (mental disorder, not otherwise specified)
Non-MDD psychiatric or neurological disorders	-	F30	130890	Date F30 first reported (manic episode)
Non-MDD psychiatric or neurological disorders	-	F31	130892	Date F31 first reported (bipolar affective disorder)
Non-MDD psychiatric or neurological disorders	-	F38	130900	Date F38 first reported (other mood [affective] disorders)
Non-MDD psychiatric or neurological disorders	-	F39	130902	Date F39 first reported (unspecified mood [affective] disorder)
Non-MDD psychiatric or neurological disorders	-	G20	131022	Date G20 first reported (parkinson's disease)
Non-MDD psychiatric or neurological disorders	-	G21	131024	Date G21 first reported (secondary parkinsonism)
Non-MDD psychiatric or neurological disorders	-	G22	131026	Date G22 first reported (parkinsonism in diseases classified elsewhere)
Non-MDD psychiatric or neurological disorders	-	G23	131028	Date G23 first reported (other degenerative diseases of basal ganglia)
Non-MDD psychiatric or neurological disorders	-	G24	131030	Date G24 first reported (dystonia)
Non-MDD psychiatric or neurological disorders	-	G25	131032	Date G25 first reported (other extrapyramidal and movement disorders)
Non-MDD psychiatric or neurological disorders	-	G26	131034	Date G26 first reported (extrapyramidal and movement disorders in diseases classified elsewhere)
Non-MDD psychiatric or neurological disorders	-	G30	131036	Date G30 first reported (alzheimer's disease)
Non-MDD psychiatric or neurological disorders	-	G31	131038	Date G31 first reported (other degenerative diseases of nervous system, not elsewhere classified)
Non-MDD psychiatric or neurological disorders	-	G32	131040	Date G32 first reported (other degenerative disorders of nervous system in diseases classified elsewhere)
Non-MDD psychiatric or neurological disorders	-	G80	131100	Date G80 first reported (infantile cerebral palsy)
Non-MDD psychiatric or neurological disorders	-	G81	131102	Date G81 first reported (hemiplegia)

Non-MDD psychiatric or neurological disorders	-	G82	131104	Date G82 first reported (paraplegia and tetraplegia)
Non-MDD psychiatric or neurological disorders	-	G83	131106	Date G83 first reported (other paralytic syndromes)
**Assessment dates**				
Baseline assessment date	0	-	53	Date of attending assessment centre
First imaging visit date	2	-	53	Date of attending assessment centre
**Baseline variables**				
Age	-	-	21022	Age at recruitment
Sex	-	-	31	Sex
Ethnicity	-	-	21000	Ethnic background
Education	0	-	6138	Qualifications
Townsend deprivation index	-	-	22189	Townsend deprivation index at recruitment
BMI	0	-	21001	Body mass index (BMI)
Smoking status	0	-	20116	Smoking status
Hypertension	-	I10	131286	Date I10 first reported (essential (primary) hypertension)
Hypertension	-	I11	131288	Date I11 first reported (hypertensive heart disease)
Hypertension	-	I12	131290	Date I12 first reported (hypertensive renal disease)
Hypertension	-	I13	131292	Date I13 first reported (hypertensive heart and renal disease)
Hypertension	-	I15	131294	Date I15 first reported (secondary hypertension)
Diabetes	-	E10	130706	Date E10 first reported (insulin-dependent diabetes mellitus)
Diabetes	-	E11	130708	Date E11 first reported (non-insulin-dependent diabetes mellitus)
Diabetes	-	E12	130710	Date E12 first reported (malnutrition-related diabetes mellitus)
Diabetes	-	E13	130712	Date E13 first reported (other specified diabetes mellitus)
Diabetes	-	E14	130714	Date E14 first reported (unspecified diabetes mellitus)
**MDD**				
MDD diagnosis	-	F32	130894	Date F32 first reported (depressive episode)
MDD diagnosis	-	F33	130896	Date F33 first reported (recurrent depressive disorder)
MDD diagnosis	-	F34	130898	Date F34 first reported (persistent mood [affective] disorders)
MDD diagnosis	-	F38	130900	Date F38 first reported (other mood [affective] disorders)
MDD diagnosis	-	F39	130902	Date F39 first reported (unspecified mood [affective] disorder)
PHQ-4	0	-	2050	Frequency of depressed mood in last 2 weeks
PHQ-4	0	-	2060	Frequency of unenthusiasm / disinterest in last 2 weeks
PHQ-4	0	-	2070	Frequency of tenseness / restlessness in last 2 weeks
PHQ-4	0	-	2080	Frequency of tiredness / lethargy in last 2 weeks
**Conditions in the Healthspan Definition**				
Death	-	Any	40000	Date of death
Cancer				
ICD-10	-	C00-C97 excl. C44	41280; 40005	Date of first in-patient diagnosis - ICD10; Date of cancer diagnosis
ICD-9	-	140-209 excl. 173	41281; 40005	Date of first in-patient diagnosis - ICD9; Date of cancer diagnosis
Diabetes	-	E10	130706	Date E10 first reported (insulin-dependent diabetes mellitus)
Diabetes	-	E11	130708	Date E11 first reported (non-insulin-dependent diabetes mellitus)
Diabetes	-	E12	130710	Date E12 first reported (malnutrition-related diabetes mellitus)
Diabetes	-	E13	130712	Date E13 first reported (other specified diabetes mellitus)
Diabetes	-	E14	130714	Date E14 first reported (unspecified diabetes mellitus)
Congestive heart failure	-	I50	131354	Date I50 first reported (heart failure)
Myocardial infarction	-	I21	131298	Date I21 first reported (acute myocardial infarction)
Myocardial infarction	-	I22	131300	Date I22 first reported (subsequent myocardial infarction)
Myocardial infarction	-	I23	131302	Date I23 first reported (certain current complications following acute myocardial infarction)
Myocardial infarction	-	I24	131304	Date I24 first reported (other acute ischaemic heart diseases)
Myocardial infarction	-	I25	131306	Date I25 first reported (chronic ischaemic heart disease)
Stroke	-	I60	131360	Date I60 first reported (subarachnoid haemorrhage)
Stroke	-	I61	131362	Date I61 first reported (intracerebral haemorrhage)
Stroke	-	I62	131364	Date I62 first reported (other nontraumatic intracranial haemorrhage)

Stroke	-	I63	131366	Date I63 first reported (cerebral infarction)
Stroke	-	I64	131368	Date I64 first reported (stroke, not specified as haemorrhage or infarction)
COPD	-	J40	131484	Date J40 first reported (bronchitis, not specified as acute or chronic)
COPD	-	J41	131486	Date J41 first reported (simple and mucopurulent chronic bronchitis)
COPD	-	J42	131488	Date J42 first reported (unspecified chronic bronchitis)
COPD	-	J43	131490	Date J43 first reported (emphysema)
COPD	-	J44	131492	Date J44 first reported (other chronic obstructive pulmonary disease)
Dementia	-	F00	130836	Date F00 first reported (dementia in alzheimer's disease)
Dementia	-	F01	130838	Date F01 first reported (vascular dementia)
Dementia	-	F02	130840	Date F02 first reported (dementia in other diseases classified elsewhere)
Dementia	-	F03	130842	Date F03 first reported (unspecified dementia)
Dementia	-	G30	131036	Date G30 first reported (alzheimer's disease)
Dementia	-	G31	131038	Date G31 first reported (other degenerative diseases of nervous system, not elsewhere classified)
**MDD adverse outcomes**				
Dementia	-	F00	130836	Date F00 first reported (dementia in alzheimer's disease)
Dementia	-	F01	130838	Date F01 first reported (vascular dementia)
Dementia	-	F02	130840	Date F02 first reported (dementia in other diseases classified elsewhere)
Dementia	-	F03	130842	Date F03 first reported (unspecified dementia)
Dementia	-	G30	131036	Date G30 first reported (alzheimer's disease)
Dementia	-	G31	131038	Date G31 first reported (other degenerative diseases of nervous system, not elsewhere classified)
Death	-	Any	40000	Date of death
**Proteomic aging measures**				
Proteins	0	-	30900	Number of proteins measured
**Cognitive function**				
Cognitive function	0	-	20023	Mean time to correctly identify matches
Cognitive function	0	-	20016	Fluid intelligence / reasoning
Cognitive function	2	-	23324	Number of symbol digit matches made correctly
Cognitive function	2	-	6350	Duration to complete alphanumeric path (trail #2)
Cognitive function	2	-	6373	Matrix pattern completion
**IDP**				
Amygdala	2		25888/25889	Volume of grey matter in Amygdala
Angular Gyrus	2		25822/25823	Volume of grey matter in Angular Gyrus
Brain-Stem	2		25892	Volume of grey matter in Brain-Stem
Caudate	2		25880/25881	Volume of grey matter in Caudate
Central Opercular Cortex	2		25864/25865	Volume of grey matter in Central Opercular Cortex
Crus I Cerebellum	2		25900/25902	Volume of grey matter in Crus I Cerebellum
Crus II Cerebellum	2		25903/25905	Volume of grey matter in Crus II Cerebellum
Vermis Crus II Cerebellum	2		25904	Volume of grey matter in Vermis Crus II Cerebellum
Vermis Crus I Cerebellum	2		25901	Volume of grey matter in Vermis Crus I Cerebellum
I-IV Cerebellum	2		25893/25894	Volume of grey matter in I-IV Cerebellum
IX Cerebellum	2		25915/25917	Volume of grey matter in IX Cerebellum
Vermis IX Cerebellum	2		25916	Volume of grey matter in Vermis IX Cerebellum
V Cerebellum	2		25895/25896	Volume of grey matter in V Cerebellum
VI Cerebellum	2		25897/25899	Volume of grey matter in VI Cerebellum
VIIb Cerebellum	2		25906/25908	Volume of grey matter in VIIb Cerebellum
Vermis VIIb Cerebellum	2		25907	Volume of grey matter in Vermis VIIb Cerebellum
VIIIa Cerebellum	2		25909/25911	Volume of grey matter in VIIIa Cerebellum
Vermis VIIIa Cerebellum	2		25910	Volume of grey matter in Vermis VIIIa Cerebellum
VIIIb Cerebellum	2		25912/25914	Volume of grey matter in VIIIb Cerebellum
Vermis VIIIb Cerebellum	2		25913	Volume of grey matter in Vermis VIIIb Cerebellum
Vermis VI Cerebellum	2		25898	Volume of grey matter in Vermis VI Cerebellum
X Cerebellum	2		25918/25920	Volume of grey matter in X Cerebellum

Vermis X Cerebellum	2	25919	Volume of grey matter in Vermis X Cerebellum
Cingulate Gyrus, anterior division	2	25838/25839	Volume of grey matter in Cingulate Gyrus, anterior division
Cingulate Gyrus, posterior division	2	25840/25841	Volume of grey matter in Cingulate Gyrus, posterior division
Cuneal Cortex	2	25844/25845	Volume of grey matter in Cuneal Cortex
Frontal Pole	2	25782/25783	Volume of grey matter in Frontal Pole
Frontal Medial Cortex	2	25830/25831	Volume of grey matter in Frontal Medial Cortex
Frontal Operculum Cortex	2	25862/25863	Volume of grey matter in Frontal Operculum Cortex
Frontal Orbital Cortex	2	25846/25847	Volume of grey matter in Frontal Orbital Cortex
Heschl's Gyrus (includes H1 and H2)	2	25870/25871	Volume of grey matter in Heschl's Gyrus (includes H1 and H2)
Hippocampus	2	25886/25887	Volume of grey matter in Hippocampus
Inferior Frontal Gyrus, pars opercularis	2	25792/25793	Volume of grey matter in Inferior Frontal Gyrus, pars opercularis
Inferior Frontal Gyrus, pars triangularis	2	25790/25791	Volume of grey matter in Inferior Frontal Gyrus, pars triangularis
Inferior Temporal Gyrus, anterior division	2	25808/25809	Volume of grey matter in Inferior Temporal Gyrus, anterior division
Inferior Temporal Gyrus, posterior division	2	25810/25811	Volume of grey matter in Inferior Temporal Gyrus, posterior division
Inferior Temporal Gyrus, temporooccipital part	2	25812/25813	Volume of grey matter in Inferior Temporal Gyrus, temporooccipital part
Insular Cortex	2	25784/25785	Volume of grey matter in Insular Cortex
Intracalcarine Cortex	2	25828/25829	Volume of grey matter in Intracalcarine Cortex
Juxtapositional Lobule Cortex (formerly Supplementary Motor Cortex)	2	25832/25833	Volume of grey matter in Juxtapositional Lobule Cortex (formerly Supplementary Motor Cortex)
Lateral Occipital Cortex, inferior division	2	25826/25827	Volume of grey matter in Lateral Occipital Cortex, inferior division
Lateral Occipital Cortex, superior division	2	25824/25825	Volume of grey matter in Lateral Occipital Cortex, superior division
Lingual Gyrus	2	25852/25853	Volume of grey matter in Lingual Gyrus
Middle Frontal Gyrus	2	25788/25789	Volume of grey matter in Middle Frontal Gyrus
Middle Temporal Gyrus, anterior division	2	25802/25803	Volume of grey matter in Middle Temporal Gyrus, anterior division
Middle Temporal Gyrus, posterior division	2	25804/25805	Volume of grey matter in Middle Temporal Gyrus, posterior division
Middle Temporal Gyrus, temporooccipital part	2	25806/25807	Volume of grey matter in Middle Temporal Gyrus, temporooccipital part
Occipital Fusiform Gyrus	2	25860/25861	Volume of grey matter in Occipital Fusiform Gyrus
Occipital Pole	2	25876/25877	Volume of grey matter in Occipital Pole
Pallidum	2	25884/25885	Volume of grey matter in Pallidum
Paracingulate Gyrus	2	25836/25837	Volume of grey matter in Paracingulate Gyrus
Parahippocampal Gyrus, anterior division	2	25848/25849	Volume of grey matter in Parahippocampal Gyrus, anterior division
Parahippocampal Gyrus, posterior division	2	25850/25851	Volume of grey matter in Parahippocampal Gyrus, posterior division
Parietal Operculum Cortex	2	25866/25867	Volume of grey matter in Parietal Operculum Cortex
Planum Polare	2	25868/25869	Volume of grey matter in Planum Polare
Planum Temporale	2	25872/25873	Volume of grey matter in Planum Temporale
Postcentral Gyrus	2	25814/25815	Volume of grey matter in Postcentral Gyrus
Precentral Gyrus	2	25794/25795	Volume of grey matter in Precentral Gyrus
Precuneous Cortex	2	25842/25843	Volume of grey matter in Precuneous Cortex
Putamen	2	25882/25883	Volume of grey matter in Putamen
Subcallosal Cortex	2	25834/25835	Volume of grey matter in Subcallosal Cortex
Superior Frontal Gyrus	2	25786/25787	Volume of grey matter in Superior Frontal Gyrus
Superior Parietal Lobule	2	25816/25817	Volume of grey matter in Superior Parietal Lobule
Supracalcarine Cortex	2	25874/25875	Volume of grey matter in Supracalcarine Cortex
Supramarginal Gyrus, anterior division	2	25818/25819	Volume of grey matter in Supramarginal Gyrus, anterior division
Supramarginal Gyrus, posterior division	2	25820/25821	Volume of grey matter in Supramarginal Gyrus, posterior division
Superior Temporal Gyrus, anterior division	2	25798/25799	Volume of grey matter in Superior Temporal Gyrus, anterior division
Superior Temporal Gyrus, posterior division	2	25800/25801	Volume of grey matter in Superior Temporal Gyrus, posterior division
Temporal Fusiform Cortex, anterior division	2	25854/25855	Volume of grey matter in Temporal Fusiform Cortex, anterior division
Temporal Fusiform Cortex, posterior division	2	25856/25857	Volume of grey matter in Temporal Fusiform Cortex, posterior division
Temporal Occipital Fusiform Cortex	2	25858/25859	Volume of grey matter in Temporal Occipital Fusiform Cortex
Temporal Pole	2	25796/25797	Volume of grey matter in Temporal Pole
Thalamus	2	25878/25879	Volume of grey matter in Thalamus
Ventral Striatum	2	25890/25891	Volume of grey matter in Ventral Striatum

Vol of accumbens	2		25023/25024	Volume of accumbens (from T1 brain image)
Vol of amygdala	2		25021/25022	Volume of amygdala (from T1 brain image)
Vol of caudate	2		25013/25014	Volume of caudate (from T1 brain image)
Vol of hippocampus	2		25019/25020	Volume of hippocampus (from T1 brain image)
Vol of pallidum	2		25017/25018	Volume of pallidum (from T1 brain image)
Vol of putamen	2		25015/25016	Volume of putamen (from T1 brain image)
Vol of thalamus	2		25011/25012	Volume of thalamus (from T1 brain image)
Total Vol of Peri-Ventricular WMH	2		24485	Total volume of peri-ventricular white matter hyperintensities
Total Vol of Deep WMH	2		24486	Total volume of deep white matter hyperintensities
Whole Brain	2		26518	Volume of TotalGray in the whole brain generated by subcortical volumetric segmentation (aseg)
Total Vol of WMH	2		25781	Total volume of white matter hyperintensities (from T1 and T2_FLAIR images)
**PAC, HPS, and the brain proteomic aging clock GWAS**				
Genetically determined European	-	-	22026	Genetic ethnic grouping
Genotyped data	-	-	22418	Genotype calls
Imputed genotype data	-	-	22828	Imputation from genotype (WTCHG)
Poor genotype quality	-	-	22027	Outliers for heterozygosity or missing rate
Top genetic principal components 1-20	-	-	22009	Genetic principal components
Age	-	-	21022	Age at recruitment
Sex	-	-	31	Sex
Baseline assessment center	0	-	54	UK Biobank assessment centre
Genotyping array	-	-	22000	Genotype measurement batch
