# Supplementary material for: Biological Aging Acceleration in Major Depressive Disorder: A Multi‐Omics Analysis
Source: Aging Cell. 2025 Dec 4;25(1):e70310. doi: 10.1111/acel.70310 (PMC12741235; doi:10.1111/acel.70310)
Supplement: Supplementary file 2 — Table S2: acel70310‐sup‐0002‐TableS2.pdf. [file ACEL-25-e70310-s010.pdf]

**Table S2. A list of antidepressant Anatomical Therapeutic Chemical (ATC) codes and the corresponding UK Biobank codes in the baseline self-reported medication data to identify participants using antidepressants at baseline assessment**

| Category | UK Biobank Coding | Medication ATC code | Drug Class | Drug Name |
|---|---|---|---|---|
| imipramine | 1140879630 | N06AA02 | Non-selective monoamine reuptake inhibitors | Imipramine |
| clomipramine | 1140879620 | N06AA04 | Non-selective monoamine reuptake inhibitors | Clomipramine |
| anafranil 10mg capsule | 1140867690 | N06AA04 | Non-selective monoamine reuptake inhibitors | Clomipramine |
| trimipramine | 1140867756 | N06AA06 | Non-selective monoamine reuptake inhibitors | Trimipramine |
| surmontil 10mg tablet | 1140867758 | N06AA06 | Non-selective monoamine reuptake inhibitors | Trimipramine |
| lofepramine | 1140867726 | N06AA07 | Non-selective monoamine reuptake inhibitors | Lofepramine |
| amitriptyline | 1140879616 | N06AA09 | Non-selective monoamine reuptake inhibitors | Amitriptyline |
| nortriptyline | 1140867818 | N06AA10 | Non-selective monoamine reuptake inhibitors | Nortriptyline |
| allegron 10mg tablet | 1140867820 | N06AA10 | Non-selective monoamine reuptake inhibitors | Nortriptyline |
| doxepin | 1140867640 | N06AA12 | Non-selective monoamine reuptake inhibitors | Doxepin |
| dosulepin | 1140909806 | N06AA16 | Non-selective monoamine reuptake inhibitors | Dosulepin |
| dothiepin | 1140879628 | N06AA16 | Non-selective monoamine reuptake inhibitors | Dosulepin |
| prothiaden 25mg capsule | 1140867624 | N06AA16 | Non-selective monoamine reuptake inhibitors | Dosulepin |
| fluoxetine | 1140879540 | N06AB03 | Selective serotonin reuptake Inhibitors | Fluoxetine |
| prozac 20mg capsule | 1140867876 | N06AB03 | Selective serotonin reuptake Inhibitors | Fluoxetine |
| oxactin 20mg capsule | 1141174756 | N06AB03 | Selective serotonin reuptake Inhibitors | Fluoxetine |
| citalopram | 1140921600 | N06AB04 | Selective serotonin reuptake Inhibitors | Citalopram |
| cipramil 10mg tablet | 1141151946 | N06AB04 | Selective serotonin reuptake Inhibitors | Citalopram |
| paroxetine | 1140867888 | N06AB05 | Selective serotonin reuptake Inhibitors | Paroxetine |
| seroxat 20mg tablet | 1140882236 | N06AB05 | Selective serotonin reuptake Inhibitors | Paroxetine |
| sertraline | 1140867878 | N06AB06 | Selective serotonin reuptake Inhibitors | Sertraline |
| lustral 50mg tablet | 1140867884 | N06AB06 | Selective serotonin reuptake Inhibitors | Sertraline |
| fluvoxamine | 1140879544 | N06AB08 | Selective serotonin reuptake Inhibitors | Fluvoxamine |
| escitalopram | 1141180212 | N06AB10 | Selective serotonin reuptake Inhibitors | Escitalopram |
| cipralex 5mg tablet | 1141190158 | N06AB10 | Selective serotonin reuptake Inhibitors | Escitalopram |
| venlafaxine | 1140916282 | N06AX16 | Other | Venlafaxine |
| efexor 37.5mg tablet | 1140916288 | N06AX16 | Other | Venlafaxine |
| cymbalta 30mg gastro-resistant capsule | 1141201834 | N06AX21 | Other | Duloxetine |

| | | | | |
|---|---|---|---|---|
| yentreve 20mg gastro-resistant capsule | 1141200570 | N06AX21 | Other | Duloxetine |
| duloxetine | 1141200564 | N06AX21 | Other | Duloxetine |
| phenelzine | 1140867850 | N06AF03 | Monoamine oxidase inhibitors, non-selective | Phenelzine |
| tranylcypromine | 1140867914 | N06AF04 | Monoamine oxidase inhibitors, non-selective | Tranylcypromine |
| moclobemide | 1140867920 | N06AG02 | Monoamine oxidase A inhibitors | Moclobemide |
| tryptophan product | 1140867960 | N06AX02 | Other | Tryptophan |
| trazodone | 1140879634 | N06AX05 | Other | Trazodone |
| mirtazapine | 1141152732 | N06AX11 | Other | Mirtazapine |
| zispin 30mg tablet | 1141152736 | N06AX11 | Other | Mirtazapine |
| zyban 150mg m/r tablet | 1141176858 | N06AX12 | Other | Bupropion |
| bupropion | 1141176854 | N06AX12 | Other | Bupropion |
| st john's wort/hypericum [ctsu] | 1201 | N06AX25 | Other | St. John'S Wort |
