# Supplementary material for: Biological Aging Acceleration in Major Depressive Disorder: A Multi‐Omics Analysis
Source: Aging Cell. 2025 Dec 4;25(1):e70310. doi: 10.1111/acel.70310 (PMC12741235; doi:10.1111/acel.70310)
Supplement: Supplementary file 3 — Table S3: acel70310‐sup‐0003‐TableS3.pdf. [file ACEL-25-e70310-s003.pdf]

Table S3. Independent loci associated with PAC, HPS, and the brain proteomic aging clock at the 5x10^(-8) significance level after adjusting for covariates

Covariates: age, age^2, sex, age × sex, age^2 × sex, the top 20 genetic principal components, array type (BiLEVE or Axiom), and baseline assessment center

Proteomic Aging Measure	CHR	BP (GRCh37)	SNP	ALLELE0	ALLELE1	A1FREQ	N	BETA	SE	F-Statistic
BRAIN	1	156615114	rs2365715	G	A	0.61075	43707	-0.05416	0.00703	59.2889994
BRAIN	1	175569047	rs2235256	G	A	0.55829	43707	-0.05428	0.00666	66.3406502
BRAIN	4	72866018	rs187638689	G	A	0.9965	43707	-0.3535	0.06347	31.0239336
BRAIN	6	31333939	rs7761068	G	A	0.61294	43707	0.04328	0.00669	41.8369254
BRAIN	6	31603591	rs2261033	G	A	0.57228	43707	0.04113	0.00658	39.0764883
BRAIN	6	32007790	rs6477	G	C	0.87921	43707	0.06525	0.01031	40.0317276
BRAIN	6	32362245	6:32362245_TAA_T	T	TAA	0.85158	43707	0.05365	0.0092	33.9814746
BRAIN	6	32626537	rs28672722	T	G	0.77739	43707	0.04491	0.00784	32.8041338
BRAIN	6	32737494	rs6457661	T	C	0.61039	43707	0.03822	0.00668	32.7500262
BRAIN	7	123875391	rs185622583	A	C	0.97054	43707	0.11914	0.02012	35.0775496
BRAIN	7	124136563	rs10954032	C	T	0.72235	43707	0.10554	0.00732	207.739591
BRAIN	7	124153623	rs182813319	T	C	0.99388	43707	0.30877	0.04953	38.8677078
BRAIN	7	124387943	rs2107930	A	G	0.74416	43707	-0.04741	0.00754	39.570716
BRAIN	7	145363182	rs6979892	C	G	0.2865	43707	0.06146	0.00724	72.1501456
BRAIN	13	92450219	rs2989992	T	C	0.73507	43707	-0.07501	0.00742	102.250613
BRAIN	18	72209543	rs17817077	A	G	0.60708	43707	0.04608	0.00671	47.1144208
BRAIN	19	23907630	rs4449070	T	G	0.75725	43707	-0.05125	0.0077	44.3504749
BRAIN	19	45411941	rs429358	C	T	0.8391	43707	-0.06468	0.00879	54.1919941
HPS	1	11844330	rs10864543	T	C	0.40422	43,707	-0.04186	0.00659	40.3382446
HPS	4	7536364	rs4689137	G	C	0.71564	43,707	-0.03958	0.00718	30.3796546
HPS	5	156423486	5:156423486_TA_T	T	TA	0.69965	43,707	-0.06299	0.00724	75.7420506
HPS	6	31019476	rs9295948	T	G	0.87867	43,707	-0.05672	0.00987	32.9956171
HPS	6	31523676	6:31523676_TC_T	T	TC	0.77781	43,707	-0.04928	0.00804	37.5950409
HPS	9	136145404	rs9411377	A	C	0.70409	43,707	0.05039	0.0072	48.9214186
HPS	11	102705047	11:102705047_CAG_C	C	CAG	0.87302	43,707	-0.05535	0.00973	32.3417861
HPS	20	23859319	rs34269359	A	G	0.82321	43,707	-0.0595	0.00844	49.6562264
PAC	6	31019476	rs9295948	T	G	0.87867	43,707	0.06557	0.00995	43.4570616
PAC	6	31331517	rs9266345	A	G	0.61259	43,707	0.04969	0.00668	55.4110817
PAC	6	32361612	rs17208769	C	T	0.85294	43,707	0.05894	0.00925	40.6448796
PAC	6	32626451	rs28414666	A	G	0.78995	43,707	0.05247	0.00797	43.3629997
PAC	9	136149098	rs8176645	A	T	0.74017	43,707	-0.04592	0.00801	32.877322
PAC	13	46709389	rs4942487	C	T	0.08512	43,707	-0.06679	0.01164	32.9328842

PAC	19	42231159	rs9621	A	G	0.95724	43,707	-0.10213	0.016	40.7267536
