# Supplementary material for: Biological Aging Acceleration in Major Depressive Disorder: A Multi‐Omics Analysis
Source: Aging Cell. 2025 Dec 4;25(1):e70310. doi: 10.1111/acel.70310 (PMC12741235; doi:10.1111/acel.70310)
Supplement: Supplementary file 4 — Table S4: acel70310‐sup‐0004‐TableS4.pdf. [file ACEL-25-e70310-s001.pdf]

Table S4. Independent loci associated with MDD at the 1x10^(-8) significance level after adjusting for covariates

CHR	BP	SNP	EA	NEA	BETA	SE	P	FCAS	FCON	IMPINFO	NEFF	NCAS	NCON	HETI	HETDF	HETPVAL
1	154142265	rs12028949	C	T	-0.031903549	0.0049	9.26E-11	0.115	0.128	0.994	918162.24	344289	1135940	12.6	24	0.2841
1	181597915	rs80223426	G	T	0.037604022	0.0064	3.28E-09	0.0621	0.0576	0.995	967078.32	357636	1281936	37	25	0.03125
1	197822130	rs1964654	T	G	0.021595137	0.0035	5.49E-10	0.486	0.479	0.985	864889.52	322163	1190635	7.3	24	0.3586
1	198436945	rs6690989	T	C	0.046501834	0.0074	3.47E-10	0.0559	0.0595	0.971	805881.8	305809	1008389	36.6	18	0.05653
1	214423797	rs12116805	A	T	-0.020396604	0.0035	3.80E-09	0.273	0.264	0.984	967078.32	357636	1281936	20.5	25	0.175
1	217082208	rs1025651	C	T	0.020302502	0.0035	5.13E-09	0.352	0.336	0.987	943460.54	349879	1257213	0	24	0.8575
1	227004367	rs6426547	G	A	-0.019702832	0.0031	1.73E-10	0.469	0.473	0.989	967078.32	357636	1281936	1.2	25	0.4449
1	241806189	rs628720	C	T	-0.020702832	0.0036	7.84E-09	0.355	0.352	0.958	943460.54	349879	1257213	24.5	24	0.1326
1	34730150	rs771398	C	T	0.021595137	0.0037	3.48E-09	0.282	0.271	0.975	967078.32	357636	1281936	0	25	0.7314
1	37197319	rs4653218	T	C	0.020400488	0.0031	6.53E-11	0.428	0.405	0.986	967078.32	357636	1281936	0	25	0.7748
1	50377775	rs55830141	A	G	0.02709947	0.004	1.14E-11	0.222	0.22	0.955	967078.32	357636	1281936	2.4	25	0.4277
1	72729142	rs1620977	A	G	0.023599332	0.0035	2.44E-11	0.27	0.257	0.975	943460.54	349879	1257213	21.4	24	0.1676
1	72883303	rs11209963	A	G	-0.031397792	0.0041	1.08E-14	0.187	0.188	0.976	943460.54	349879	1257213	0	24	0.6559
1	73816564	rs1160682	C	T	0.030897711	0.0032	5.10E-22	0.371	0.361	0.994	967078.32	357636	1281936	22.7	25	0.1487
1	74023504	rs1604347	A	G	0.020302502	0.0033	7.82E-10	0.316	0.315	0.99	967078.32	357636	1281936	2.7	25	0.4247
1	80857014	rs1365753	C	G	0.021898469	0.0037	2.44E-09	0.282	0.278	0.98	967078.32	357636	1281936	8.7	25	0.3364
10	106599009	rs2496014	T	C	0.021497269	0.0032	1.89E-11	0.36	0.346	0.991	967078.32	357636	1281936	17.9	25	0.2074
10	106677341	rs2864034	A	C	-0.029202266	0.0038	1.67E-14	0.2	0.215	0.995	967078.32	357636	1281936	8.3	25	0.3434
11	112917378	rs12277922	T	C	0.025199801	0.0039	6.74E-11	0.225	0.224	0.988	873468.26	331205	1053466	0	19	0.4704
11	113262900	rs12360992	A	C	0.023003382	0.0031	6.00E-14	0.476	0.476	0.993	967078.32	357636	1281936	0	25	0.9546
11	113350620	rs35277073	G	C	-0.034198147	0.0033	1.68E-24	0.353	0.357	0.992	883559.9	334212	1089716	0	24	0.9517
11	113436072	rs7125588	G	A	-0.019294956	0.0031	6.30E-10	0.409	0.406	0.989	967078.32	357636	1281936	14	25	0.2607
11	113451765	rs7940164	G	T	0.025004759	0.0033	6.31E-14	0.344	0.343	0.957	943460.54	349879	1257213	14.5	24	0.2577
11	133370326	rs73025662	T	G	-0.023299334	0.0039	3.54E-09	0.194	0.204	0.986	943460.54	349879	1257213	31.6	24	0.06722
11	133834104	rs612823	C	T	-0.021203208	0.0032	4.83E-11	0.413	0.431	0.965	943460.54	349879	1257213	1.6	24	0.4393
11	16291213	rs61881745	C	T	0.025102285	0.0042	2.96E-09	0.161	0.143	0.983	967078.32	357636	1281936	0	25	0.8327
11	28591587	rs7127383	C	T	-0.024600117	0.0034	3.85E-13	0.398	0.4	0.986	943460.54	349879	1257213	39.5	24	0.02306
11	29600756	rs326756	C	T	0.020400488	0.0035	5.51E-09	0.319	0.319	0.984	967078.32	357636	1281936	11.3	25	0.299
11	32927778	rs145678014	T	G	-0.048098403	0.0082	3.91E-09	0.0392	0.0401	0.958	967078.32	357636	1281936	0	25	0.6494
11	46231891	rs76400970	T	C	0.028597175	0.0049	4.86E-09	0.12	0.125	0.98	918162.24	344289	1135940	0	24	0.572
11	48239731	rs76416526	T	C	0.032699496	0.0056	6.27E-09	0.0811	0.0832	0.994	967078.32	357636	1281936	43.7	25	0.009744
11	57450720	rs488769	C	A	0.022895877	0.0032	1.02E-12	0.345	0.344	0.991	967078.32	357636	1281936	27.9	25	0.09437
11	61610750	rs174584	A	G	0.020704182	0.0032	1.55E-10	0.356	0.369	0.997	967078.32	357636	1281936	3.3	25	0.4151
11	63859751	rs1807304	G	A	-0.025605029	0.0036	1.39E-12	0.247	0.251	0.967	967078.32	357636	1281936	7.7	25	0.352
11	73309421	rs7930907	A	G	0.025999076	0.004	1.26E-10	0.174	0.179	0.983	967078.32	357636	1281936	16.2	25	0.23
12	121207147	rs12579868	T	C	0.018596017	0.0031	1.55E-09	0.467	0.451	0.988	967078.32	357636	1281936	0	25	0.7084

12	201242	rs7135697	G	A	-0.01899935	0.0032	2.21E-09	0.467	0.466	0.979	936106.18	347935	1222884	16.7	20	0.2421
12	2345295	rs1006737	A	G	0.023003382	0.0032	7.23E-13	0.337	0.332	1	967078.32	357636	1281936	13.9	25	0.2631
12	24007901	rs4635171	G	A	-0.029099308	0.0048	1.72E-09	0.113	0.114	0.99	967078.32	357636	1281936	0	25	0.8056
12	24170368	rs6487370	T	C	-0.024098039	0.0034	1.85E-12	0.338	0.347	0.985	967078.32	357636	1281936	0	25	0.5928
12	60987918	rs2455855	T	C	-0.018398214	0.0031	4.12E-09	0.387	0.385	0.997	967078.32	357636	1281936	26.8	25	0.1048
13	54048078	rs1373285	A	G	0.023696995	0.0035	8.16E-12	0.339	0.343	0.985	967078.32	357636	1281936	38	25	0.02699
13	60228658	rs9538492	C	T	-0.024600117	0.0039	1.73E-10	0.276	0.262	0.925	967078.32	357636	1281936	5	25	0.3906
13	67596668	rs7995526	C	T	0.028801234	0.005	6.84E-09	0.106	0.105	0.997	956986.66	354629	1245686	14.1	20	0.275
13	94051114	rs2762111	T	C	0.019204409	0.0032	2.41E-09	0.343	0.342	0.999	959723.96	355692	1247607	0	21	0.8786
14	103367667	rs3783385	A	G	0.022602625	0.0037	1.30E-09	0.222	0.223	0.992	943460.54	349879	1257213	0	24	0.6604
14	104079924	rs2896488	A	G	-0.02200024	0.0036	8.61E-10	0.342	0.35	0.982	781371.12	298739	998415	0	23	0.6911
14	42097937	rs1950829	A	G	0.021996298	0.0031	5.67E-13	0.502	0.503	0.994	967078.32	357636	1281936	8.7	25	0.3368
14	75130235	rs1045430	T	G	-0.019203207	0.0031	3.41E-10	0.482	0.483	0.99	967078.32	357636	1281936	0	25	0.6935
14	75288697	rs17183201	G	T	0.037796623	0.0059	2.03E-10	0.0733	0.0724	0.979	967078.32	357636	1281936	4.4	25	0.3989
15	38869666	rs17651741	A	G	0.024204689	0.0039	3.65E-10	0.193	0.182	0.997	967078.32	357636	1281936	0	25	0.9026
15	47685306	rs1025145	G	A	0.021595137	0.0033	6.50E-11	0.331	0.329	0.986	943460.54	349879	1257213	43.7	24	0.01106
15	48009093	rs7166621	C	T	-0.020896826	0.0034	4.37E-10	0.396	0.398	0.976	859942.12	326455	1064993	22.3	23	0.1617
15	66393606	rs7174069	T	C	0.026797712	0.0039	7.68E-12	0.207	0.2	0.972	933368.9	346872	1220963	0	19	0.9248
15	78079425	rs4420498	T	C	-0.019702832	0.0033	1.87E-09	0.414	0.415	0.99	967078.32	357636	1281936	0	25	0.9003
15	91426560	rs4702	G	A	0.024799924	0.0032	4.72E-15	0.445	0.446	0.974	940260	348949	1251483	16.2	23	0.2381
16	21614009	rs2369818	T	C	0.020096702	0.0035	8.76E-09	0.431	0.427	0.958	943460.54	349879	1257213	0	24	0.6746
16	60730282	rs28711200	T	G	0.017997077	0.0031	9.62E-09	0.507	0.498	0.987	918162.24	344289	1135940	0	24	0.8218
16	6328922	rs17219976	A	G	0.029704423	0.0045	5.53E-11	0.153	0.159	0.949	967078.32	357636	1281936	39.5	25	0.02137
17	31542491	rs389265	T	C	-0.022695608	0.0038	1.40E-09	0.258	0.27	0.971	967078.32	357636	1281936	23.4	25	0.1404
17	65825354	rs12601921	T	C	0.024204689	0.0038	3.01E-10	0.2	0.197	0.987	967078.32	357636	1281936	4.8	25	0.3939
17	77810623	rs62075210	T	C	-0.026600682	0.0046	5.95E-09	0.144	0.152	0.945	943460.54	349879	1257213	0	24	0.5939
18	26569901	rs9964679	A	G	0.022103899	0.0034	1.54E-10	0.322	0.329	0.987	859942.12	326455	1064993	0	23	0.5343
18	31300559	rs1941686	G	A	-0.023504069	0.0034	2.47E-12	0.462	0.46	0.981	943460.54	349879	1257213	0	24	0.7926
18	35188449	rs58011088	A	G	0.026203665	0.0035	1.26E-13	0.334	0.336	0.982	943460.54	349879	1257213	0	24	0.9521
18	39294665	rs17656030	G	A	-0.032998511	0.0048	7.55E-12	0.112	0.106	0.994	967078.32	357636	1281936	24.3	25	0.1302
18	50755757	rs62100777	A	G	0.023902056	0.0031	7.62E-15	0.464	0.471	0.99	967078.32	357636	1281936	32.2	25	0.05915
18	50762502	rs7231742	G	A	-0.027895486	0.0048	6.09E-09	0.137	0.152	0.953	841507.98	323949	804611	0	23	0.5531
18	52471537	rs12968035	G	A	-0.028296599	0.004	7.83E-13	0.206	0.226	0.978	943460.54	349879	1257213	0	24	0.5393
18	52754086	rs55943003	A	G	0.025199801	0.0036	3.69E-12	0.239	0.228	0.989	943460.54	349879	1257213	0	24	0.9269
18	52902683	rs59231191	T	C	0.029296632	0.0051	6.62E-09	0.105	0.0961	0.995	943460.54	349879	1257213	0	24	0.831
18	53099012	rs12967143	G	C	0.027702712	0.0034	4.36E-16	0.294	0.277	0.985	967078.32	357636	1281936	15.8	25	0.2365
18	53207207	rs1452787	G	A	0.021702782	0.0034	1.38E-10	0.284	0.28	0.996	967078.32	357636	1281936	0	25	0.6955
18	53250324	rs79703067	C	T	0.078099282	0.0126	5.60E-10	0.0161	0.0137	0.96	966213.12	357151	1281310	0	25	0.5324
18	77578191	rs11663602	A	C	0.020704182	0.0035	2.15E-09	0.285	0.293	0.988	943460.54	349879	1257213	0	24	0.8724

18	77627876	rs9958792	T	C	-0.025399863	0.0042	1.02E-09	0.178	0.165	0.977	943460.54	349879	1257213	0	24	0.9382
2	104412924	rs72820274	A	G	0.018703985	0.0031	2.38E-09	0.417	0.42	0.988	943460.54	349879	1257213	17.6	24	0.2151
2	125052996	rs780024	A	T	0.020900064	0.0033	1.54E-10	0.345	0.351	0.987	943460.54	349879	1257213	0	24	0.4734
2	148993376	rs78716456	A	G	-0.034995253	0.006	6.00E-09	0.0978	0.0988	0.881	841507.98	323949	804611	0	23	0.6046
2	157145541	rs11903494	T	G	-0.027802945	0.0038	4.91E-13	0.205	0.202	0.99	943460.54	349879	1257213	0	24	0.4903
2	162033854	rs1267079	C	T	-0.019804827	0.0033	2.34E-09	0.304	0.298	0.997	967078.32	357636	1281936	19.5	25	0.1873
2	175239185	rs17255455	A	C	-0.021295141	0.0031	1.18E-11	0.429	0.429	0.994	943460.54	349879	1257213	26.4	24	0.1131
2	185822703	rs1429428	C	T	0.023804413	0.0033	1.05E-12	0.305	0.302	0.989	967078.32	357636	1281936	33.3	25	0.05182
2	208076936	rs10187826	G	A	0.023696995	0.0035	6.34E-12	0.325	0.322	0.986	967078.32	357636	1281936	31.2	25	0.06643
2	22554648	rs67446571	G	A	0.02489747	0.0032	3.12E-15	0.386	0.388	0.986	967078.32	357636	1281936	0	25	0.573
2	22888130	rs13012903	A	G	0.023101103	0.0032	4.61E-13	0.368	0.368	0.987	967078.32	357636	1281936	13.9	25	0.2625
2	233667744	rs283466	G	A	0.019096496	0.0032	2.03E-09	0.396	0.392	0.987	943460.54	349879	1257213	27.2	24	0.1052
2	57987593	rs11682175	C	T	0.021095908	0.0031	4.81E-12	0.461	0.447	0.996	967078.32	357636	1281936	35.5	25	0.03904
2	58969692	rs55899910	T	A	0.058599042	0.0093	3.49E-10	0.0282	0.0259	0.983	966761.6	357511	1281720	0	25	0.9821
20	44688665	rs9074	A	G	0.024204689	0.0034	1.84E-12	0.265	0.261	0.998	967078.32	357636	1281936	0	25	0.5175
20	45840459	rs6063085	C	A	-0.024702613	0.0036	4.32E-12	0.371	0.373	0.967	918162.24	344289	1135940	7.7	24	0.3524
20	45854487	rs3091566	C	T	0.025004759	0.0043	7.62E-09	0.172	0.166	0.964	967078.32	357636	1281936	37.1	25	0.03085
20	51205902	rs17805843	T	A	-0.024497632	0.0041	1.75E-09	0.178	0.185	0.987	943460.54	349879	1257213	0	24	0.754
22	41408754	rs13056300	C	T	0.02709947	0.0035	1.71E-14	0.268	0.27	0.979	943460.54	349879	1257213	22.8	24	0.1512
22	46458123	rs7292297	G	T	-0.021203208	0.0033	1.71E-10	0.463	0.457	0.971	967078.32	357636	1281936	0	25	0.793
3	115069703	rs73230700	T	C	0.05209897	0.0084	5.80E-10	0.0371	0.0407	0.975	921086.58	342749	1215938	18.2	20	0.223
3	117517339	rs9842435	A	G	-0.029397915	0.0046	1.18E-10	0.138	0.149	0.966	967078.32	357636	1281936	17.2	25	0.2176
3	117943952	rs4688087	A	G	0.02350166	0.0038	4.26E-10	0.213	0.207	0.986	967078.32	357636	1281936	36	25	0.03644
3	157899116	rs1724729	T	C	-0.019702832	0.0031	2.04E-10	0.42	0.42	0.992	967078.32	357636	1281936	0	25	0.9101
3	16862555	rs7617959	C	G	0.023198815	0.0034	1.42E-11	0.375	0.374	0.987	788235.26	301823	859306	0	23	0.6651
3	18809536	rs4364183	G	A	-0.023995606	0.0036	3.47E-11	0.289	0.3	0.984	943460.54	349879	1257213	5.2	24	0.389
3	193413502	rs12630	C	T	-0.017695649	0.0031	6.75E-09	0.482	0.481	0.992	967078.32	357636	1281936	0	25	0.4807
3	44241830	rs35875034	T	G	0.020704182	0.0033	2.50E-10	0.479	0.475	0.989	943460.54	349879	1257213	25.4	24	0.1224
3	44464619	rs12054231	T	C	0.023198815	0.0037	3.47E-10	0.332	0.334	0.973	841507.98	323949	804611	0	23	0.7078
3	48731487	rs3172494	T	G	-0.032698827	0.0048	1.45E-11	0.122	0.129	0.959	943460.54	349879	1257213	0	24	0.4812
3	49621994	rs7629322	C	T	0.034101865	0.0042	3.78E-16	0.163	0.152	0.986	967078.32	357636	1281936	5.2	25	0.3882
3	51874679	rs16943	C	T	-0.023196982	0.0036	1.07E-10	0.328	0.342	0.964	943460.54	349879	1257213	0	24	0.7671
3	52540773	rs1010553	C	T	0.018596017	0.0031	1.52E-09	0.473	0.466	0.986	967078.32	357636	1281936	5.4	25	0.3849
3	71814431	rs116310555	C	T	0.027196792	0.0047	8.24E-09	0.125	0.125	0.973	967078.32	357636	1281936	1.7	25	0.4391
3	76211681	rs11128505	C	A	-0.019203207	0.0031	7.76E-10	0.478	0.47	0.988	943460.54	349879	1257213	37.8	24	0.0301
4	115522306	rs13134858	A	G	0.027196792	0.0046	3.55E-09	0.146	0.155	0.953	967078.32	357636	1281936	23.3	25	0.1419
4	123388893	rs45534736	G	C	-0.042197941	0.0069	8.53E-10	0.0512	0.0512	0.995	967078.32	357636	1281936	0	25	0.8796
4	140910964	rs7696858	A	G	0.023296517	0.0031	3.31E-14	0.461	0.473	0.992	967078.32	357636	1281936	0	25	0.7774
4	80217741	rs1484145	T	C	0.023804413	0.0033	2.61E-13	0.477	0.486	0.993	967078.32	357636	1281936	0	25	0.5021

5	103723455	rs1530303	T	C	0.029102387	0.0032	2.12E-19	0.346	0.341	0.987	967078.32	357636	1281936	35.2	25	0.04041
5	120049338	rs7714971	A	G	0.025199801	0.0035	2.90E-13	0.269	0.281	0.991	967078.32	357636	1281936	8.9	25	0.334
5	124262051	rs2408225	C	T	-0.019702832	0.0032	6.41E-10	0.447	0.469	0.985	918162.24	344289	1135940	4.3	24	0.4018
5	124980248	rs1602401	G	A	-0.020600746	0.0033	3.20E-10	0.351	0.357	0.982	943460.54	349879	1257213	17.5	24	0.2173
5	143877756	rs358667	C	T	-0.019702832	0.0034	5.99E-09	0.299	0.297	0.985	956986.66	354629	1245686	25.4	20	0.1411
5	153215007	rs1993739	T	C	-0.024395157	0.0042	6.45E-09	0.167	0.169	0.993	918162.24	344289	1135940	0	24	0.5022
5	166989513	rs4044321	A	G	0.02399969	0.0034	1.65E-12	0.353	0.353	0.988	967078.32	357636	1281936	13	25	0.2751
5	167140568	rs10475523	G	C	0.040997979	0.0071	7.26E-09	0.056	0.059	0.941	943460.54	349879	1257213	22.3	24	0.1569
5	30839451	rs4626350	G	A	-0.023104874	0.0031	7.62E-14	0.456	0.458	0.99	967078.32	357636	1281936	0	25	0.5167
5	61514611	rs12520974	T	C	-0.023504069	0.0031	3.25E-14	0.481	0.488	0.99	943460.54	349879	1257213	25.5	24	0.1222
6	101248480	rs9322213	C	T	0.023003382	0.0031	5.51E-14	0.502	0.517	0.991	967078.32	357636	1281936	24.1	25	0.133
6	152215199	rs9479138	T	G	0.030703778	0.0033	1.15E-20	0.351	0.353	0.983	943460.54	349879	1257213	19.8	24	0.187
6	165068701	rs12174848	A	T	0.022602625	0.0039	6.51E-09	0.228	0.235	0.969	967078.32	357636	1281936	27.8	25	0.09534
6	26393021	rs1614887	G	A	0.019704583	0.0031	1.99E-10	0.471	0.487	0.995	943460.54	349879	1257213	14.1	24	0.2622
6	28705074	rs1311911	T	G	-0.049705083	0.006	1.80E-16	0.0912	0.0843	1	920386.24	342971	1203151	55.9	19	0.001266
6	39965946	rs1923466	C	A	-0.02220471	0.0035	2.17E-10	0.309	0.316	0.985	967078.32	357636	1281936	18.7	25	0.1975
6	64621869	rs386409	C	T	-0.020396604	0.0035	3.48E-09	0.271	0.261	0.988	967078.32	357636	1281936	0	25	0.857
7	108987009	rs576121532	G	T	0.120898179	0.021	8.66E-09	0.00742	0.0119	0.907	796760.5	291760	1111628	0	15	0.5268
7	110212702	rs1396172	A	G	0.019204409	0.0031	9.95E-10	0.436	0.44	0.987	967078.32	357636	1281936	0	25	0.5912
7	114059156	rs2894699	T	C	-0.020202707	0.0032	2.34E-10	0.43	0.428	0.986	918162.24	344289	1135940	0	24	0.717
7	114946569	rs10233079	G	T	0.024497472	0.0042	5.14E-09	0.171	0.171	0.97	967078.32	357636	1281936	0	25	0.6212
7	12263538	rs10950392	C	T	0.021301503	0.0031	7.40E-12	0.409	0.404	0.994	967078.32	357636	1281936	7.2	25	0.3584
7	135119417	rs2551774	A	G	0.022103899	0.0031	1.41E-12	0.401	0.401	0.99	967078.32	357636	1281936	0	25	0.6083
7	2078855	rs55683212	C	G	-0.030799472	0.0038	2.15E-16	0.209	0.208	0.997	967078.32	357636	1281936	44.7	25	0.007845
7	3390546	rs7804333	C	T	0.021497269	0.0034	2.83E-10	0.285	0.291	0.994	967078.32	357636	1281936	9.7	25	0.322
7	3521803	rs10499337	T	C	-0.026600682	0.0036	3.09E-13	0.23	0.234	0.988	967078.32	357636	1281936	19.5	25	0.1877
7	82445482	rs2214415	G	A	0.021095908	0.003	4.41E-12	0.492	0.492	0.993	967078.32	357636	1281936	12.8	25	0.2786
8	14218081	rs28897173	C	T	-0.021795812	0.0034	1.26E-10	0.285	0.275	0.988	967078.32	357636	1281936	0	25	0.6096
8	14628926	rs2256035	A	G	-0.019294956	0.0032	9.30E-10	0.391	0.389	0.986	967078.32	357636	1281936	0	25	0.8807
8	63385433	rs74524106	T	C	-0.088405075	0.0148	2.19E-09	0.0129	0.0152	0.894	939822.1	348189	1237399	18.2	20	0.2227
8	65562019	rs7837935	T	G	-0.026795823	0.0044	1.24E-09	0.143	0.143	0.988	967078.32	357636	1281936	0	25	0.7348
8	94583638	rs4236809	A	G	-0.021295141	0.0034	5.95E-10	0.338	0.35	0.987	967078.32	357636	1281936	0	25	0.7832
9	108904396	rs117723312	G	C	0.076998077	0.0122	2.57E-10	0.0189	0.0186	0.926	967078.32	357636	1281936	35	25	0.04167
9	11109859	rs7875617	A	G	-0.027895486	0.0042	4.64E-11	0.179	0.176	0.979	841507.98	323949	804611	50.4	23	0.002697
9	11274206	rs10756218	C	T	-0.023504069	0.0034	8.50E-12	0.392	0.381	0.979	943460.54	349879	1257213	2.3	24	0.4293
9	119629708	rs4837958	G	A	0.018998382	0.0032	4.55E-09	0.473	0.472	0.991	967078.32	357636	1281936	0	25	0.5138
9	119731359	rs2418449	C	T	-0.022398995	0.0034	4.15E-11	0.283	0.287	0.996	956986.66	354629	1245686	35	20	0.05854
9	122657987	rs2151642	A	T	-0.020396604	0.0034	2.47E-09	0.384	0.383	0.986	934683.94	343048	1258126	33.1	21	0.06718
9	127818276	rs589292	T	C	-0.019600848	0.0034	8.77E-09	0.291	0.286	0.99	967078.32	357636	1281936	0	25	0.8328

9	31246469	rs10970237	G	A	-0.019498873	0.0032	1.94E-09	0.337	0.35	0.99	967078.32	357636	1281936	0	25	0.5141
9	37025418	rs3758178	G	A	0.025004759	0.0035	1.24E-12	0.404	0.414	0.982	894544.46	336532	1111217	0	23	0.8121
9	37377466	rs62533809	C	T	0.028402795	0.0041	4.59E-12	0.18	0.183	0.98	943460.54	349879	1257213	41.8	24	0.01581
9	96225478	rs7023933	T	A	0.029500548	0.0043	1.07E-11	0.168	0.167	0.981	841507.98	323949	804611	0	23	0.8501
9	96343060	rs10821163	C	G	-0.02110107	0.0036	4.07E-09	0.348	0.361	0.977	792355.68	301059	1019916	15.7	22	0.2476
9	98243868	rs80155616	T	C	0.029500548	0.0051	7.72E-09	0.1	0.108	0.988	967078.32	357636	1281936	33.2	25	0.05228

#CHROM: Chromosome
POS: Base-pair position (GRCh37)
ID: Marker ID (reference SNP cluster ID or chromosome-position ID)
EA: SNP effect allele for freq and ln(OR)
NEA: SNP non-effect allele
BETA: ln(Odds Ratio) effect of EA
SE: standard error of ln(OR)
PVAL: P-value, uncorrected
FCAS: frequency of EA in cases
FCON: frequency of EA in controls
IMPINFO: imputation INFO score
NEFF: effective sample size
NCAS: total sample size cases
NCON: total sample size controls
HETI: heterogeneity I^2 statistic
HETDF: heterogeneity test degrees-of-freedom
HETPVAL: heterogeneity test P-value
