# Supplementary material for: Biological Aging Acceleration in Major Depressive Disorder: A Multi‐Omics Analysis
Source: Aging Cell. 2025 Dec 4;25(1):e70310. doi: 10.1111/acel.70310 (PMC12741235; doi:10.1111/acel.70310)
Supplement: Supplementary file 5 — Table S5: acel70310‐sup‐0005‐TableS5.pdf. [file ACEL-25-e70310-s005.pdf]

Table S5. Descriptive summary of baseline participant characteristics in the observational association cohort of the UK Biobank (n=50,297)

**Variable**	**Mean (SD); Median (Min, Max); n (%)**
Age (Years)	57 (8); 58 (39, 70)
Sex	
Female	27,195 (54%)
Male	23,102 (46%)
Ethnicity	
White	47,237 (94%)
Black	1,137 (2.3%)
Asian	1,114 (2.2%)
Other	809 (1.6%)
Education	
None of the above	8,875 (18%)
Other professional qualifications eg: nursing, teaching	2,686 (5.3%)
NVQ or HND or HNC or equivalent	3,385 (6.7%)
CSEs or equivalent	2,738 (5.4%)
O levels/GCSEs or equivalent	10,571 (21%)
A levels/AS levels or equivalent	5,633 (11%)
College or University degree	16,409 (33%)
Townsend Deprivation Index	-1.2 (3.2); -2.1 (-6.3, 10.4)
Smoking Status	
Never	27,355 (54%)
Current	5,301 (11%)
Previous	17,641 (35%)
BMI	27.5 (4.8); 26.8 (14.3, 69.0)
MDD Status (=Yes)	4,477 (8.9%)
Diabetes Status (=Yes)	2,774 (5.5%)
Hypertention Status (=Yes)	14,031 (28%)
PHQ-4 (=Positive)	10,920 (24%)
Antidepressant Use (=Yes)	3,902 (7.8%)
**Proteomic Aginc Clocks**	
Systemic	
PAC	53 (11); 52 (19, 114)
HPS	0.77 (0.18); 0.82 (0.00, 0.98)
Organ-Specific	
Adipose	0.04 (0.37); 0.02 (-2.00, 3.15)
Artery	0.01 (0.72); 0.00 (-3.45, 4.56)
Brain	0.07 (0.93); 0.03 (-3.41, 5.83)
Heart	0.00 (0.48); -0.01 (-2.90, 2.64)
Immune	0.01 (0.78); -0.06 (-3.94, 4.81)
Intestine	0.04 (0.69); 0.00 (-2.62, 4.87)
Kidney	0.07 (0.60); 0.00 (-2.85, 4.89)
Liver	-0.04 (0.74); -0.10 (-3.50, 5.27)
