# Supplementary material for: Biological Aging Acceleration in Major Depressive Disorder: A Multi‐Omics Analysis
Source: Aging Cell. 2025 Dec 4;25(1):e70310. doi: 10.1111/acel.70310 (PMC12741235; doi:10.1111/acel.70310)
Supplement: Supplementary file 6 — Table S6: acel70310‐sup‐0006‐TableS6.pdf. [file ACEL-25-e70310-s008.pdf]

Table S6. Descriptive summary of baselien participant characteristics by MDD status at baseline in the observational association cohort of the UK Biobank (n=50,297)

[1] Mean (SD); Median (Minimum, Maximum) or Frequency (%);
[2]Wilcoxon rank sum tests for continuou variables; chi-squared tests for categorical variables

	Baseline MDD		
**Variable**	**No, N = 45820 (91%)[1]**	**Yes, N = 4477 (8.9%)[1]**	**P-Value[2]**
Age (Years)	57 (8); 58 (39, 70)	55 (8); 56 (40, 70)	<0.001
Sex			<0.001
Female	24,293 (53%)	2,902 (65%)	
Male	21,527 (47%)	1,575 (35%)	
Ethnicity			<0.001
White	42,959 (94%)	4,278 (96%)	
Black	1,084 (2.4%)	53 (1.2%)	
Asian	1,050 (2.3%)	64 (1.4%)	
Other	727 (1.6%)	82 (1.8%)	
Education			<0.001
None of the above	7,996 (17%)	879 (20%)	
Other professional qualifications eg: nursing, teaching	2,445 (5.3%)	241 (5.4%)	
NVQ or HND or HNC or equivalent	3,098 (6.8%)	287 (6.4%)	
CSEs or equivalent	2,466 (5.4%)	272 (6.1%)	
O levels/GCSEs or equivalent	9,631 (21%)	940 (21%)	
A levels/AS levels or equivalent	5,110 (11%)	523 (12%)	
College or University degree	15,074 (33%)	1,335 (30%)	
Townsend Deprivation Index	-1.3 (3.1); -2.1 (-6.3, 10.4)	-0.6 (3.4); -1.5 (-6.3, 10.1)	<0.001
Smoking Status			<0.001
Never	25,262 (55%)	2,093 (47%)	
Current	4,548 (9.9%)	753 (17%)	
Previous	16,010 (35%)	1,631 (36%)	
BMI	27.4 (4.7); 26.7 (14.3, 69.0)	28.2 (5.4); 27.4 (15.8, 59.2)	<0.001
Diabetes Status (=Yes)	2,491 (5.4%)	283 (6.3%)	0.013
Hypertention Status (=Yes)	12,651 (28%)	1,380 (31%)	<0.001
PHQ-4 (=Positive)	8,674 (21%)	2,246 (56%)	<0.001
Antidepressant Use (=Yes)	1,633 (3.6%)	2,269 (51%)	<0.001
