# Supplementary material for: Biological Aging Acceleration in Major Depressive Disorder: A Multi‐Omics Analysis
Source: Aging Cell. 2025 Dec 4;25(1):e70310. doi: 10.1111/acel.70310 (PMC12741235; doi:10.1111/acel.70310)
Supplement: Supplementary file 7 — Table S7: acel70310‐sup‐0007‐TableS7.pdf. [file ACEL-25-e70310-s009.pdf]

Table S7. Spearman correlations between the residuals of HPS, PAC, the brain-specific proteomic aging clock, and brain MRI image-derived phenotypes (IDPs).

FDR-adjusted *p* -values < 0.05 are **highlighted in red**. Correlations **> 0.07** are **highlighted in yellow**, while correlations < **-0.07** are **highlighted in green**.

						Rho			FDR-Adjusted P-Value		
Group	IDP Description	Method	Position	Field(s)	n	HPS	PAC	Brain	HPS	PAC	Brain
Regional GMV	Volume of grey matter in Amygdala	FAST	L/R	25888/25889	5768	0.067	-0.051	-0.060	0.000	0.000	0.000
Regional GMV	Volume of grey matter in Angular Gyrus	FAST	L/R	25822/25823	5768	0.022	-0.027	-0.014	0.122	0.049	0.318
Regional GMV	Volume of grey matter in Brain-Stem	FAST		25892	5768	-0.006	-0.012	-0.004	0.714	0.384	0.800
Regional GMV	Volume of grey matter in Caudate	FAST	L/R	25880/25881	5768	0.100	-0.079	-0.060	0.000	0.000	0.000
Regional GMV	Volume of grey matter in Central Opercular Cortex	FAST	L/R	25864/25865	5768	0.074	-0.070	-0.096	0.000	0.000	0.000
Regional GMV	Volume of grey matter in Crus I Cerebellum	FAST	L/R	25900/25902	5768	0.051	-0.047	-0.052	0.000	0.001	0.000
Regional GMV	Volume of grey matter in Crus II Cerebellum	FAST	L/R	25903/25905	5768	0.056	-0.018	-0.036	0.000	0.192	0.012
Regional GMV	Volume of grey matter in Vermis Crus II Cerebellum	FAST		25904	5768	0.031	-0.012	-0.026	0.031	0.384	0.066
Regional GMV	Volume of grey matter in Vermis Crus I Cerebellum	FAST		25901	5768	-0.019	0.007	0.016	0.195	0.617	0.262
Regional GMV	Volume of grey matter in I-IV Cerebellum	FAST	L/R	25893/25894	5768	0.024	-0.025	-0.017	0.090	0.074	0.225
Regional GMV	Volume of grey matter in IX Cerebellum	FAST	L/R	25915/25917	5768	0.108	-0.059	-0.069	0.000	0.000	0.000
Regional GMV	Volume of grey matter in Vermis IX Cerebellum	FAST		25916	5768	0.067	-0.034	-0.047	0.000	0.015	0.001
Regional GMV	Volume of grey matter in V Cerebellum	FAST	L/R	25895/25896	5768	0.033	-0.026	-0.029	0.020	0.067	0.042
Regional GMV	Volume of grey matter in VI Cerebellum	FAST	L/R	25897/25899	5768	0.056	-0.040	-0.051	0.000	0.005	0.000
Regional GMV	Volume of grey matter in VIIb Cerebellum	FAST	L/R	25906/25908	5768	0.061	-0.020	-0.039	0.000	0.146	0.005
Regional GMV	Volume of grey matter in Vermis VIIb Cerebellum	FAST		25907	5768	0.067	-0.039	-0.059	0.000	0.005	0.000
Regional GMV	Volume of grey matter in VIIIa Cerebellum	FAST	L/R	25909/25911	5768	0.098	-0.051	-0.066	0.000	0.000	0.000
Regional GMV	Volume of grey matter in Vermis VIIIa Cerebellum	FAST		25910	5768	0.075	-0.047	-0.060	0.000	0.001	0.000
Regional GMV	Volume of grey matter in VIIIb Cerebellum	FAST	L/R	25912/25914	5768	0.103	-0.064	-0.068	0.000	0.000	0.000
Regional GMV	Volume of grey matter in Vermis VIIIb Cerebellum	FAST		25913	5768	0.073	-0.044	-0.047	0.000	0.002	0.001
Regional GMV	Volume of grey matter in Vermis VI Cerebellum	FAST		25898	5768	0.044	-0.031	-0.034	0.002	0.027	0.017
Regional GMV	Volume of grey matter in X Cerebellum	FAST	L/R	25918/25920	5768	-0.006	-0.009	0.003	0.711	0.530	0.828
Regional GMV	Volume of grey matter in Vermis X Cerebellum	FAST		25919	5768	0.078	-0.050	-0.046	0.000	0.000	0.001
Regional GMV	Volume of grey matter in Cingulate Gyrus, anterior division	FAST	L/R	25838/25839	5768	0.029	-0.014	-0.034	0.040	0.312	0.016
Regional GMV	Volume of grey matter in Cingulate Gyrus, posterior division	FAST	L/R	25840/25841	5768	0.019	-0.028	-0.010	0.195	0.047	0.465
Regional GMV	Volume of grey matter in Cuneal Cortex	FAST	L/R	25844/25845	5768	0.016	-0.038	-0.032	0.277	0.007	0.021
Regional GMV	Volume of grey matter in Frontal Pole	FAST	L/R	25782/25783	5768	0.049	-0.062	-0.070	0.001	0.000	0.000
Regional GMV	Volume of grey matter in Frontal Medial Cortex	FAST	L/R	25830/25831	5768	0.018	-0.032	-0.016	0.203	0.024	0.256

Regional GMV	Volume of grey matter in Frontal Operculum Cortex	FAST	L/R	25862/25863	5768	0.022	-0.028	-0.051	0.134	0.047	0.000
Regional GMV	Volume of grey matter in Frontal Orbital Cortex	FAST	L/R	25846/25847	5768	0.040	-0.060	-0.054	0.006	0.000	0.000
Regional GMV	Volume of grey matter in Heschl's Gyrus (includes H1 and H2)	FAST	L/R	25870/25871	5768	0.064	-0.057	-0.078	0.000	0.000	0.000
Regional GMV	Volume of grey matter in Hippocampus	FAST	L/R	25886/25887	5768	0.030	-0.025	-0.017	0.034	0.074	0.225
Regional GMV	Volume of grey matter in Inferior Frontal Gyrus, pars opercularis	FAST	L/R	25792/25793	5768	0.027	-0.025	-0.033	0.057	0.070	0.020
Regional GMV	Volume of grey matter in Inferior Frontal Gyrus, pars triangularis	FAST	L/R	25790/25791	5768	0.000	-0.004	-0.014	0.993	0.784	0.308
Regional GMV	Volume of grey matter in Inferior Temporal Gyrus, anterior division	FAST	L/R	25808/25809	5768	0.027	-0.037	-0.034	0.056	0.008	0.015
Regional GMV	Volume of grey matter in Inferior Temporal Gyrus, posterior division	FAST	L/R	25810/25811	5768	0.017	-0.023	-0.021	0.239	0.092	0.136
Regional GMV	Volume of grey matter in Inferior Temporal Gyrus, temporooccipital part	FAST	L/R	25812/25813	5768	-0.034	0.021	0.017	0.017	0.128	0.224
Regional GMV	Volume of grey matter in Insular Cortex	FAST	L/R	25784/25785	5768	0.075	-0.062	-0.087	0.000	0.000	0.000
Regional GMV	Volume of grey matter in Intracalcarine Cortex	FAST	L/R	25828/25829	5768	0.032	-0.043	-0.031	0.027	0.002	0.028
Regional GMV	Volume of grey matter in Juxtapositional Lobule Cortex (formerly Supplementary Motor Cortex)	FAST	L/R	25832/25833	5768	0.029	-0.041	-0.045	0.041	0.004	0.001
Regional GMV	Volume of grey matter in Lateral Occipital Cortex, inferior division	FAST	L/R	25826/25827	5768	0.027	-0.048	-0.031	0.056	0.001	0.028
Regional GMV	Volume of grey matter in Lateral Occipital Cortex, superior division	FAST	L/R	25824/25825	5768	0.068	-0.075	-0.061	0.000	0.000	0.000
Regional GMV	Volume of grey matter in Lingual Gyrus	FAST	L/R	25852/25853	5768	0.010	-0.031	-0.028	0.501	0.025	0.045
Regional GMV	Volume of grey matter in Middle Frontal Gyrus	FAST	L/R	25788/25789	5768	0.036	-0.039	-0.040	0.012	0.005	0.004
Regional GMV	Volume of grey matter in Middle Temporal Gyrus, anterior division	FAST	L/R	25802/25803	5768	0.026	-0.049	-0.044	0.066	0.000	0.002
Regional GMV	Volume of grey matter in Middle Temporal Gyrus, posterior division	FAST	L/R	25804/25805	5768	0.049	-0.050	-0.055	0.001	0.000	0.000
Regional GMV	Volume of grey matter in Middle Temporal Gyrus, temporooccipital part	FAST	L/R	25806/25807	5768	0.012	-0.019	-0.009	0.407	0.166	0.548
Regional GMV	Volume of grey matter in Occipital Fusiform Gyrus	FAST	L/R	25860/25861	5768	0.038	-0.054	-0.044	0.008	0.000	0.002
Regional GMV	Volume of grey matter in Occipital Pole	FAST	L/R	25876/25877	5768	0.001	-0.034	-0.022	0.969	0.018	0.124
Regional GMV	Volume of grey matter in Pallidum	FAST	L/R	25884/25885	5768	0.123	-0.090	-0.087	0.000	0.000	0.000
Regional GMV	Volume of grey matter in Paracingulate Gyrus	FAST	L/R	25836/25837	5768	0.055	-0.067	-0.069	0.000	0.000	0.000
Regional GMV	Volume of grey matter in Parahippocampal Gyrus, anterior division	FAST	L/R	25848/25849	5768	0.000	-0.009	-0.005	0.982	0.529	0.748
Regional GMV	Volume of grey matter in Parahippocampal Gyrus, posterior division	FAST	L/R	25850/25851	5768	0.011	-0.034	-0.006	0.452	0.016	0.667
Regional GMV	Volume of grey matter in Parietal Operculum Cortex	FAST	L/R	25866/25867	5768	0.021	-0.029	-0.045	0.153	0.041	0.001
Regional GMV	Volume of grey matter in Planum Polare	FAST	L/R	25868/25869	5768	0.076	-0.060	-0.082	0.000	0.000	0.000
Regional GMV	Volume of grey matter in Planum Temporale	FAST	L/R	25872/25873	5768	0.020	-0.031	-0.047	0.155	0.025	0.001
Regional GMV	Volume of grey matter in Postcentral Gyrus	FAST	L/R	25814/25815	5768	0.080	-0.073	-0.074	0.000	0.000	0.000
Regional GMV	Volume of grey matter in Precentral Gyrus	FAST	L/R	25794/25795	5768	0.074	-0.074	-0.072	0.000	0.000	0.000
Regional GMV	Volume of grey matter in Precuneous Cortex	FAST	L/R	25842/25843	5768	0.037	-0.049	-0.046	0.010	0.000	0.001
Regional GMV	Volume of grey matter in Putamen	FAST	L/R	25882/25883	5768	0.050	-0.032	-0.041	0.000	0.023	0.003
Regional GMV	Volume of grey matter in Subcallosal Cortex	FAST	L/R	25834/25835	5768	0.043	-0.042	-0.057	0.002	0.003	0.000
Regional GMV	Volume of grey matter in Superior Frontal Gyrus	FAST	L/R	25786/25787	5768	0.039	-0.050	-0.050	0.007	0.000	0.000

Regional GMV	Volume of grey matter in Superior Parietal Lobule	FAST	L/R	25816/25817	5768	0.041	-0.051	-0.057	0.004	0.000	0.000
Regional GMV	Volume of grey matter in Supracalcarine Cortex	FAST	L/R	25874/25875	5768	0.014	-0.030	-0.016	0.336	0.030	0.262
Regional GMV	Volume of grey matter in Supramarginal Gyrus, anterior division	FAST	L/R	25818/25819	5768	0.043	-0.039	-0.046	0.002	0.005	0.001
Regional GMV	Volume of grey matter in Supramarginal Gyrus, posterior division	FAST	L/R	25820/25821	5768	0.025	-0.042	-0.027	0.088	0.003	0.054
Regional GMV	Volume of grey matter in Superior Temporal Gyrus, anterior division	FAST	L/R	25798/25799	5768	0.035	-0.049	-0.053	0.016	0.000	0.000
Regional GMV	Volume of grey matter in Superior Temporal Gyrus, posterior division	FAST	L/R	25800/25801	5768	0.054	-0.056	-0.063	0.000	0.000	0.000
Regional GMV	Volume of grey matter in Temporal Fusiform Cortex, anterior division	FAST	L/R	25854/25855	5768	0.034	-0.044	-0.039	0.016	0.002	0.006
Regional GMV	Volume of grey matter in Temporal Fusiform Cortex, posterior division	FAST	L/R	25856/25857	5768	0.023	-0.028	-0.035	0.110	0.043	0.014
Regional GMV	Volume of grey matter in Temporal Occipital Fusiform Cortex	FAST	L/R	25858/25859	5768	0.008	-0.014	-0.024	0.584	0.328	0.096
Regional GMV	Volume of grey matter in Temporal Pole	FAST	L/R	25796/25797	5768	0.003	-0.045	-0.022	0.828	0.002	0.123
Regional GMV	Volume of grey matter in Thalamus	FAST	L/R	25878/25879	5768	-0.004	0.002	0.018	0.773	0.868	0.220
Regional GMV	Volume of grey matter in Ventral Striatum	FAST	L/R	25890/25891	5768	0.066	-0.046	-0.064	0.000	0.001	0.000
Subcortical Vol	Volume of accumbens (from T1 brain image)	FIRST	L/R	25023/25024	5768	0.052	-0.070	-0.059	0.000	0.000	0.000
Subcortical Vol	Volume of amygdala (from T1 brain image)	FIRST	L/R	25021/25022	5768	-0.018	-0.004	0.010	0.211	0.760	0.502
Subcortical Vol	Volume of caudate (from T1 brain image)	FIRST	L/R	25013/25014	5768	0.047	-0.026	-0.026	0.001	0.059	0.069
Subcortical Vol	Volume of hippocampus (from T1 brain image)	FIRST	L/R	25019/25020	5768	0.035	-0.054	-0.031	0.015	0.000	0.028
Subcortical Vol	Volume of pallidum (from T1 brain image)	FIRST	L/R	25017/25018	5768	0.035	-0.032	-0.054	0.016	0.023	0.000
Subcortical Vol	Volume of putamen (from T1 brain image)	FIRST	L/R	25015/25016	5768	0.006	-0.012	-0.002	0.698	0.384	0.883
Subcortical Vol	Volume of thalamus (from T1 brain image)	FIRST	L/R	25011/25012	5768	0.066	-0.068	-0.056	0.000	0.000	0.000
Regional and Tissue Vol	Total volume of peri-ventricular white matter hyperintensities			24485	5577	0.115	-0.114	-0.075	0.000	0.000	0.000
Regional and Tissue Vol	Total volume of deep white matter hyperintensities			24486	5577	0.261	-0.273	-0.209	0.000	0.000	0.000
Regional and Tissue Vol	Volume of TotalGray in the whole brain generated by subcortical volumetric segmentation (aseg)	aseg		26518	5702	0.065	-0.072	-0.073	0.000	0.000	0.000
Regional and Tissue Vol	Total volume of white matter hyperintensities (from T1 and T2_FLAIR images)	BIANCA		25781	5577	0.145	-0.147	-0.104	0.000	0.000	0.000
