# Supplementary material for: Biological Aging Acceleration in Major Depressive Disorder: A Multi‐Omics Analysis
Source: Aging Cell. 2025 Dec 4;25(1):e70310. doi: 10.1111/acel.70310 (PMC12741235; doi:10.1111/acel.70310)
Supplement: Supplementary file 8 — Table S8: acel70310‐sup‐0008‐TableS8.pdf. [file ACEL-25-e70310-s007.pdf]

Table S8. Mendelian randomization results

			IVW							MR-PRESSO				MR-RAP			
Exposure	Outcome	#SNPs	BETA	95% CI LB	95% CI UB	P-Value	Q	Q P-Value	Mean F	BETA	95% CI LB	95% CI UB	P-Value	BETA	95% CI LB	95% CI UB	P-Value
MDD	PAC	159	0.059	0.004	0.115	0.035	207.79	0.005	43.79	0.059	0.004	0.115	0.037	0.061	0.012	0.110	0.015
MDD	HPS	159	-0.096	-0.149	-0.042	0.000	196.09	0.021	43.79	-0.096	-0.149	-0.042	0.001	-0.099	-0.147	-0.050	0.000
MDD	BRAIN	159	0.089	0.033	0.145	0.002	213.53	0.002	43.79	0.089	0.033	0.145	0.002	0.092	0.042	0.141	0.000
PAC	MDD	9	-0.017	-0.202	0.168	0.857	27.62	0.001	43.25	-0.017	-0.202	0.168	0.861	-0.018	-0.118	0.081	0.718
HPS	MDD	8	0.024	-0.112	0.159	0.731	11.47	0.119	43.50	0.024	-0.112	0.159	0.741	0.025	-0.083	0.133	0.655
BRAIN	MDD	18	-0.007	-0.124	0.110	0.906	62.06	0.000	56.58	-0.007	-0.124	0.110	0.908	-0.007	-0.068	0.053	0.810

			Weighted-Median					MR-Egger							
Exposure	Outcome	#SNPs	BETA	95% CI LB	95% CI UB	P-Value	BETA	95% CI LB	95% CI UB	BETA P-Value	I-square	Intercept	95% CI LB	95% CI UB	P-Value
MDD	PAC	159	0.039	-0.034	0.112	0.291	-0.060	-0.312	0.193	0.643	0.000	0.003	-0.003	0.009	0.343
MDD	HPS	159	-0.061	-0.133	0.011	0.095	0.040	-0.203	0.283	0.748	0.000	-0.003	-0.009	0.003	0.263
MDD	BRAIN	159	0.078	0.005	0.152	0.036	-0.028	-0.285	0.229	0.833	0.000	0.003	-0.003	0.009	0.362
PAC	MDD	9	-0.073	-0.226	0.081	0.352	0.403	-0.320	1.126	0.274	0.000	-0.025	-0.066	0.016	0.239
HPS	MDD	8	0.031	-0.127	0.190	0.700	-0.691	-1.407	0.024	0.058	0.070	0.037	0.000	0.074	0.047
BRAIN	MDD	18	0.003	-0.100	0.105	0.961	-0.018	-0.352	0.317	0.918	0.792	0.001	-0.020	0.021	0.947
