# Supplementary material for: Biological Aging Acceleration in Major Depressive Disorder: A Multi‐Omics Analysis
Source: Aging Cell. 2025 Dec 4;25(1):e70310. doi: 10.1111/acel.70310 (PMC12741235; doi:10.1111/acel.70310)
Supplement: Supplementary file 9 — Data S1: acel70310‐sup‐0009‐SupplementaryMethods.docx. [file ACEL-25-e70310-s004.docx]

**Supplementary Methods**

UKB is a large population-based prospective study recruiting volunteers aged 40 to 69 years between 2006 and 2010^1,2^. For the current analysis, we included 53,014 participants that had proteomic data available for the calculation of systemic and organ-specific proteomic aging measures^3^. Participants with diagnosis of psychotic disorders (e.g., schizophrenia and bipolar disorder), or neurological disorders (ICD-10 codes listed in **Supplementary Table 1**) were first excluded, followed by those with any missing baseline covariates across analyses, resulting in a final sample of 50,297.

MDD diagnosis was ascertained using the first-occurrence data released by the UKB, including multi-source data (primary care, hospital inpatient, death register, and self-reported records) based on the ICD-10 codes (**Supplementary Table 1)**. The data period varies by source and country (England, Scotland, and Wales), with over 80% of participants attending baseline assessment centers in England. Additionally, Patient Health Questionnaire-4 (PHQ-4) data were collected through four Likert-scale survey questions (field IDs in **Supplementary Table 1**) assessing the frequency of depressed mood, unenthusiasm/disinterest, tenseness/restlessness, and tiredness/lethargy^4^. To identify possible cases of mild depression or that were not readily diagnosed in the UK Biobank, we included individuals with a total PHQ-4 score of ≥3 or lifetime MDD diagnosis (broad MDD category). Additionally, current MDD episode was defined by PHQ-4 score ≥ 3, while remitted MDD was defined as participants with a lifetime diagnosis of MDD and current PHQ-4 score <3. Participants without a history of MDD were classified as never depressed.

**Biological Aging Proteomic Measures**

The proteomic aging clock (PAC) and healthspan proteomic score (HPS) are systemic proteomic biomarkers of biological aging^5,6^. They were developed using normalized protein expression (NPX) data from 2,920 proteins measured in the UKB with the Olink Explore 3072 assay to predict mortality and healthspan. Additionally, we included organ-specific proteomic clocks for eight tissues: brain, adipose, immune system, heart, arteries, intestine, kidneys, and liver^7^. For our analysis, we derived PAC, HPS, and the organ-specific proteomic clocks after imputing missing NPX data across the 2,920 proteins using a k-nearest neighbors’ approach (k=10) implemented via the R package ‘multiUS.’

**Outcomes**

We examined the association between a history of MDD and proteomic aging measures at baseline. Additionally, among participants without a history of MDD, we assessed whether baseline proteomic aging measures were associated with incident MDD during follow-up. In participants with a lifetime history of MDD, we evaluated the associations between baseline proteomic aging measures and the risk of ADRD and mortality, including deaths by suicide. The measurements for these outcomes, including disease ICD-10 codes and field IDs, are detailed in **Supplementary Table 1**.

We also examined the associations between PAC, HPS, and the brain proteomic aging clock with cognitive function and brain MRI image-derived phenotypes (IDPs) (field IDs in **Supplementary Table 1**). Cognitive function was assessed through online cognitive tests, with measurement details available elsewhere^8^. IDPs were derived from T1- and T2-weighted imaging scans, processed using an image-processing pipeline developed and run on behalf of UKB^9^. For analysis, left and right hemisphere measurements of the same tract were averaged. Additional technical details are provided in the UK Biobank Brain Imaging Documentation (<https://biobank.ndph.ox.ac.uk/showcase/refer.cgi?id=1977>, Date of access 2/27/2025).

**Covariates**

Baseline participant characteristics were selected as covariates for evidence of associations with MDD. Age, sex, ethnicity, and education were self-reported at recruitment. Townsend deprivation index was derived at the postcode level based on the preceding national census data. Higher scores represent greater levels of material deprivation. Body mass index was calculated using weight and height physically measured at baseline assessment. Smoking status was assessed via an online questionnaire. Hypertension and diabetes status at baseline were determined using the first occurrence data described previously. UKB field IDs to extract the data above are listed in **Supplementary Table 1**.

For analyses restricted to participants with a history of MDD at baseline, use of antidepressants was assessed using self-reported medication data at baseline linking UKB prescription codes to ATC codes of antidepressants (**Supplementary Table 2**)^10^.

**Statistical Methods**

We used Spearman coefficients to evaluate the correlations between chronological age and proteomic aging measures, as well as the residuals of proteomic aging measures after adjusting for chronological age. Before conducting the association analyses, PAC, HPS, and organ-specific proteomic aging measures were transformed using the rank-based inverse normal transformation into z-scores to correct distributional skewness and unify the scales. Linear regression models were used to examine the associations between MDD status and these proteomic aging measures at baseline, adjusting for chronological age and other covariates.

Residuals from linear regression models for PAC, HPS, and the brain proteomic aging clock were correlated with cognitive function and brain MRI IDPs using Spearman correlation coefficients. Models were adjusted for chronological age and the time gap between protein measurements and the cognitive function measure or IDP, if different. Additionally, for IDPs, a head size proxy was included in the adjustments, specifically volumetric scaling from the T1 head image to standard space.

Cox regression models were used to investigate the associations of baseline proteomic aging measures with disease outcomes and mortality during follow-up since recruitment. In these survival analyses, participants were censored at the date of death or the last follow-up date of hospital inpatient data (England 2022/10/31, Scotland 2022/08/31, and Wales 2022/05/31) (primary source of EHRs), whichever occurred first. P-values were adjusted for multiple testing using the Benjamini-Hochberg false discovery rate (FDR) method^11^. All hypotheses were two-sided, with significance assessed at the 5% level. Statistical analyses were performed using R version 4.4.3.

**The Essential Hypertension Epigenetics (EH-Epi) study: external validation for the association between depression and accelerated biological aging.**

**Cohort Description**

The external replication analyses were conducted in an independent sample of twins from the Finnish Twin Cohort (FTC), specifically those who participated in the Essential Hypertension Epigenetics (EH-Epi) study, a sub-study involving intensive phenotyping^12^. Same-sex twin pairs with discordant blood pressure levels were invited to participate in the EH-Epi study.

During in-person visits between 2013 and 2015, the twins provided fasting blood samples, and additional physiological and clinical measurements were collected^13^. Using the data available from the EH-Epi study, we evaluated the association between depression and biological aging acceleration based on proteomic and epigenetic clocks. Forty-four (11%) participants met the criteria for CES-D-defined MDD, and 63 (16%) reported a physician-diagnosed MDD. Broad MDD (n=75) was defined as self-reported physician-diagnosed MDD (n=63) and/or current antidepressant use (n=29).

**Proteomic Data**

Proteomic data were obtained using the Olink Explore 3072 platform (Olink Proteomics AB, Uppsala, Sweden) from plasma samples of 415 EH-Epi twins^14^. Quality control was performed according to Olink internal criteria using the R package ‘OlinkAnalyze’ (version 3.4.1), leading to the exclusion of a few outlier samples. After quality control, normalized protein expression (NPX) data were used to compute PAC, HPS, and organ-specific proteomic aging clocks for 401 twins, where missing proteins were replaced by zero, which was close to the mean across proteins.

**DNA Methylation Data**

DNA methylation levels were measured using the Infinium Illumina HumanMethylation450K array and preprocessed with the R package ‘meffil’^15^. In this study, we used six previously generated epigenetic age estimates, available for 379 of the 401 Finnish twins^16,17^. These included estimates based on Horvath^18^, Hannum^19^, DNAm PhenoAge^20^, and GrimAge^21^, calculated using their PC score versions^22^, and the GrimAge2^23^. Additionally, we computed DunedinPACE estimates^24^, which reflect individuals' pace of aging based on DNA methylation.

**Depression Assessments**

**Depression was assessed using three measures:**

- Depressive symptoms were assessed using the Center for Epidemiological Study – Depression 20 items (CES-D-20) scale, with total scores ranging from 0 to 60. A score of 20 or higher indicates a clinically relevant level of depression. Both the CES-D total score and the CES-D-defined depression status (≥20) were analyzed.
- Self-reported physician diagnosis from the 2011 questionnaire conducted 1–2 years before blood sampling.
- Broad MDD classification, defined as either a self-reported physician diagnosis or current use of antidepressant medication at the time of blood sampling.

**Statistical Methods**

We assessed the Spearman correlations between chronological age and BA measures (proteomic and epigenetic clocks), as well as their residuals after adjusting for chronological age in linear regression models. We used Generalized estimating equation (GEE) models to examine the associations between depression measures (CES-D total score, CES-D-defined MDD, lifetime history of MDD, and broad MDD category) and accelerated BA (outcome), as GEE models enable correction for non-independence of observations induced by family relatedness. Models were adjusted for chronological age and sex. Biological aging acceleration was assessed using proteomic and epigenetic clocks, which were transformed using the rank-based inverse-normal transformation to ensure comparability and correct distributional skewness. For each BA measure, p-values were adjusted for multiple testing using the Benjamini-Hochberg FDR method, with significance assessed at the 5% level.

**Bidirectional Mendelian Randomization Analysis**

We conducted a bidirectional Mendelian randomization (MR) analysis to assess the bidirectional causal relationship between MDD and proteomic aging. To evaluate the causal effect of proteomic aging on MDD, we selected genetic instruments associated with accelerated proteomic aging from genome-wide association studies (GWAS) on PAC (n=9), HPS (n=8), and the brain proteomic aging clock (n=18) (**Supplementary Table 3**). These instruments were identified in genetically determined Europeans from the UKB-PPP, after excluding individuals with poor-quality genotypes (outliers in heterogeneity or high missing rates) (n=43,707).

The GWAS were performed for PAC, HPS, and brain-specific proteomic aging clock after the inverse normal transformation in REGENIE^25^ using a two-step approach, first modeling the whole-genome genotyped data excluding variants with minor allele frequencies less than 0.01 to predict phenotypic values while accounting for population structure and relatedness, and then linking the whole-genome imputed SNPs with residual phenotypic values from Step 1 using linear regression models. Both steps 1 and 2 were adjusted for age, age², sex, age × sex, age² × sex, the top 20 genetic principal components, array type (BiLEVE or Axiom), and baseline assessment center. Linkage disequilibrium (LD) clumping using PLINK^26^ was applied to the GWAS summary statistics to identify independent loci at the genome-wide significance level (p< 5×10⁻⁸) with r² < 0.01 and a minimum distance of 250 kb between loci. Of note, the LD structure was estimated using 5,000 unrelated UKB participants of European descent and SNPs with an imputation info score (INFO)> 0.3 and a minor allele frequency >0.001 (~16 million SNPs). Genetically determined Europeans (n = 364,919), excluding individuals with poor-quality genotypes and those included in the GWAS, were used to estimate associations between the genetic instruments and MDD using Cox regression models, with the same adjustments as in the PAC, HPS, and brain proteomic aging clock GWAS. MDD (n=47,657, 13.1%) was confirmed using the first occurrence data of ICD-10 codes F32, F33, F34, F38, and F39, with individuals with negative ages at diagnosis excluded due to data errors.

For the causal effect of MDD on proteomic aging, genetic instruments for MDD (n=159) (**Supplementary Table 4**) were selected based on the LD clumping on the latest meta-GWAS summary statistics using Europeans only (310,128 cases and 1,035,355 controls), published by the Major Depressive Disorder Working Group of the Psychiatric Genomics Consortium, excluding 23andMe and UK Biobank cohorts^27^. We clumped SNPs with p ≤ 1×10^-8^, INFO ≥ 0.6 that were in linkage disequilibrium (r^2^ > 0.1) and were within 3 Mb windows of an index SNP, which resulted in a total of 159 loci. The SNP-MDD association statistics were obtained from the MDD GWAS. The association statistics of these SNPs with PAC, HPS, and the brain proteomic aging clock were obtained from the GWAS of PAC, HPS, and the brain proteomic aging clock as described above.

Two-sample Mendelian randomization (MR) analysis was conducted using the inverse variance weighting (IVW) method^28^ as the primary approach to meta-analyzing causal estimates from individual genetic instruments, with weights inversely proportional to their standard errors. The validity of MR analysis relies on three assumptions: (1) relevance—the genetic variants are strongly associated with the exposure; (2) independence—the variants are independent of confounders; and (3) exclusion restriction—the variants affect the outcome only through the exposure. To assess the robustness of our findings, we performed sensitivity analyses using additional MR methods: MR-Egger regression^29^ to detect horizontal pleiotropy, the Robust Adjusted Profile Score (MR-RAPS) method^30^ to account for residual weak instrument bias, pleiotropy, and extreme outliers, and MR-PRESSO (Mendelian Randomization Pleiotropy RESidual Sum and Outlier)^31^ to identify and correct for pleiotropic outliers. We ensured the validity of the MR findings by requiring a mean F-statistic ≥ 10, indicating strong genetic instruments, and assessing heterogeneity by Q-statistic. Additionally, we required a non-significant intercept test (p ≥ 0.01) in MR-Egger analyses, suggesting little evidence of horizontal pleiotropy. The validity of MR was confirmed by an I^2^ ≥ 0.9 to support the assumption that the genetic instrument-exposure associations were primarily due to true heterogeneity rather than random sampling error^32^. A violation of this assumption would attenuate the effect estimate and bias the pleiotropy test. All MR analyses were conducted using the R package *MendelianRandomization* v0.10.0^33^.

**References**

1. Sudlow, C.*, et al.* UK biobank: an open access resource for identifying the causes of a wide range of complex diseases of middle and old age. *PLoS Med* **12**, e1001779 (2015).

2. Bycroft, C.*, et al.* The UK Biobank resource with deep phenotyping and genomic data. *Nature* **562**, 203-209 (2018).

3. Sun, B.B.*, et al.* Plasma proteomic associations with genetics and health in the UK Biobank. *Nature*, 1-10 (2023).

4. Gao, X.*, et al.* Accelerated biological aging and risk of depression and anxiety: evidence from 424,299 UK Biobank participants. *Nat Commun* **14**, 2277 (2023).

5. Kuo, C.L.*, et al.* Proteomic aging clock (PAC) predicts age‐related outcomes in middle‐aged and older adults. *Aging Cell* **23**, e14195 (2024).

6. Kuo, C.L.*, et al.* A proteomic signature of healthspan. *medRxiv* (2024).

7. Goeminne, L.J.E.*, et al.* Plasma protein-based organ-specific aging and mortality models unveil diseases as accelerated aging of organismal systems. *Cell Metab.* (2024).

8. Fawns-Ritchie, C. & Deary, I.J. Reliability and validity of the UK Biobank cognitive tests. *PLoS One* **15**, e0231627 (2020).

9. Alfaro-Almagro, F.*, et al.* Image processing and Quality Control for the first 10,000 brain imaging datasets from UK Biobank. *Neuroimage* **166**, 400-424 (2018).

10. Cai, N.*, et al.* Minimal phenotyping yields genome-wide association signals of low specificity for major depression. *Nat Genet* **52**, 437-447 (2020).

11. Benjamini, Y. & Hochberg, Y. Controlling the False Discovery Rate: A Practical and Powerful Approach to Multiple Testing. *Journal of the Royal Statistical Society. Series B (Methodological)* **57**, 289-300 (1995).

12. Kaprio, J.*, et al.* The Older Finnish Twin Cohort - 45 Years of Follow-up. *Twin Res Hum Genet* **22**, 240-254 (2019).

13. Huang, Y.*, et al.* Identification, Heritability, and Relation With Gene Expression of Novel DNA Methylation Loci for Blood Pressure. *Hypertension* **76**, 195-205 (2020).

14. Drouard, G.*, et al.* Twin study provides heritability estimates for 2,321 plasma proteins and assesses missing SNP heritability. *medRxiv*, 2024.2004.2024.24306270 (2024).

15. Min, J.L., Hemani, G., Davey Smith, G., Relton, C. & Suderman, M. Meffil: efficient normalization and analysis of very large DNA methylation datasets. *Bioinformatics* **34**, 3983-3989 (2018).

16. Sehovic, E.*, et al.* DNA methylation sites in early adulthood characterised by pubertal timing and development: a twin study. *Clin Epigenetics* **15**, 181 (2023).

17. Kankaanpää, A.*, et al.* The role of adolescent lifestyle habits in biological aging: A prospective twin study. *Elife* **11**(2022).

18. Horvath, S. DNA methylation age of human tissues and cell types. *Genome Biol* **14**, R115 (2013).

19. Hannum, G.*, et al.* Genome-wide methylation profiles reveal quantitative views of human aging rates. *Mol Cell* **49**, 359-367 (2013).

20. Levine, M.E.*, et al.* An epigenetic biomarker of aging for lifespan and healthspan. *Aging (Albany NY)* **10**, 573-591 (2018).

21. Lu, A.T.*, et al.* DNA methylation GrimAge strongly predicts lifespan and healthspan. *Aging (Albany NY)* **11**, 303-327 (2019).

22. Higgins-Chen, A.T.*, et al.* A computational solution for bolstering reliability of epigenetic clocks: implications for clinical trials and longitudinal tracking. *Nature Aging* **2**, 644-661 (2022).

23. Piirtola, M.*, et al.* Traumatic life events as predictors for depression in middle-aged men and women: A Finnish twin study. *J Affect Disord* **370**, 470-480 (2025).

24. Belsky, D.W.*, et al.* DunedinPACE, a DNA methylation biomarker of the pace of aging. *Elife* **11**(2022).

25. Mbatchou, J.*, et al.* Computationally efficient whole-genome regression for quantitative and binary traits. *Nat. Genet.* **53**, 1097-1103 (2021).

26. Purcell, S.*, et al.* PLINK: a tool set for whole-genome association and population-based linkage analyses. *Am J Hum Genet* **81**, 559-575 (2007).

27. Consortium, M.D.D.W.G.o.t.P.G.*, et al.* Trans-ancestry genome-wide study of depression identifies 697 associations implicating cell types and pharmacotherapies. *Cell* (2025).

28. Burgess, S., Butterworth, A. & Thompson, S.G. Mendelian randomization analysis with multiple genetic variants using summarized data. *Genet Epidemiol* **37**, 658-665 (2013).

29. Bowden, J., Davey Smith, G. & Burgess, S. Mendelian randomization with invalid instruments: effect estimation and bias detection through Egger regression. *Int J Epidemiol* **44**, 512-525 (2015).

30. Zhao, Q., Wang, J., Hemani, G., Bowden, J. & Small, D.S. Statistical inference in two-sample summary-data Mendelian randomization using robust adjusted profile score. *The Annals of Statistics* **48**, 1742-1769, 1728 (2020).

31. Verbanck, M., Chen, C.Y., Neale, B. & Do, R. Detection of widespread horizontal pleiotropy in causal relationships inferred from Mendelian randomization between complex traits and diseases. *Nat Genet* **50**, 693-698 (2018).

32. Bowden, J.*, et al.* Assessing the suitability of summary data for two-sample Mendelian randomization analyses using MR-Egger regression: the role of the I2 statistic. *Int J Epidemiol* **45**, 1961-1974 (2016).

33. Yavorska, O.O. & Burgess, S. MendelianRandomization: an R package for performing Mendelian randomization analyses using summarized data. *Int J Epidemiol* **46**, 1734-1739 (2017).
