# Supplementary material for: Biological Aging Acceleration in Major Depressive Disorder: A Multi‐Omics Analysis
Source: Aging Cell. 2025 Dec 4;25(1):e70310. doi: 10.1111/acel.70310 (PMC12741235; doi:10.1111/acel.70310)
Supplement: Supplementary file 10 — Figure S1: Spearman correlations between chronological age and proteomic aging clocks: (a) chronological age and proteomic aging clocks; (b) residuals of proteomic aging clocks after adjusting for chronological age in linear regression models. Figure S2: Associations between proteomic aging (PA) measures (z‐scores) and a history of MDD at baseline after adjusting for covariates (Active: diagnosed with MDD and PHQ‐4 positive; Remitted: diagnosed with MDD and PHQ‐4 negative; Never Dep: never diagnosed with MDD). Figure S3: Associations between antidepressant use (users vs. non‐users) and proteomic aging (PA) measures (z‐scores) in participants with MDD at baseline after adjusting for covariates (MDD Diag.: diagnosed with MDD before or at baseline; Active: diagnosed and PHQ‐4 positive; Remitted: diagnosed and PHQ‐4 negative). Figure S4: Spearman correlations between the residuals of HPS, PAC, the brain proteomic aging clock, and cognitive function measures. Significance levels: p < 0.05 (*), p < 0.01 (**), p < 0.001 (***). Figure S5: Spearman correlations between the residuals of HPS, PAC, the brain proteomic aging clock, and brain MRI image‐derived phenotypes (IDPs). IDPs are labeled if the FDR‐adjusted p‐value < 0.05 and the absolute Spearman correlation > 0.07, selected to ensure meaningful effect sizes while maintaining clarity. Figure S6: Biological aging acceleration in MDD based on age group (40–59 years vs. 60 or more years) and sex (male vs. female). Figure S7: Spearman correlations between chronological age, proteomic and epigenetic aging clocks Figure S8: Spearman correlations between proteomic and epigenetic aging clocks after adjusting for chronological age in linear regression models. Figure S9: Mendelian randomization analysis for the effects of PAC, HPS, and brain proteomic aging clock on MDD. [file ACEL-25-e70310-s006.docx]

**Supplementary Figures**

**Figure S1. Spearman correlations between chronological age and proteomic aging clocks: (a) chronological age and proteomic aging clocks; (b) residuals of proteomic aging clocks after adjusting for chronological age in linear regression models**

**Figure S2. Associations between proteomic aging (PA) measures (z-scores) and a history of MDD at baseline after adjusting for covariates (Active: diagnosed with MDD and PHQ-4 positive; Remitted: diagnosed with MDD and PHQ-4 negative; Never Dep: never diagnosed with MDD)**

**Figure S3. Associations between antidepressant use (users vs. non-users) and proteomic aging (PA) measures (z-scores) in participants with MDD at baseline after adjusting for covariates (MDD Diag.: diagnosed with MDD before or at baseline; Active: diagnosed and PHQ-4 positive; Remitted: diagnosed and PHQ-4 negative)**

**Figure S4. Spearman correlations between the residuals of HPS, PAC, the brain proteomic aging clock, and cognitive function measures. Significance levels: p < 0.05 (*), p < 0.01 (**), p < 0.001 (***)**

**Figure S5. Spearman correlations between the residuals of HPS, PAC, the brain proteomic aging clock, and brain MRI image-derived phenotypes (IDPs). IDPs are labeled if the FDR-adjusted p-value < 0.05 and the absolute Spearman correlation > 0.07, selected to ensure meaningful effect sizes while maintaining clarity**

**Figure S6. Biological aging acceleration in MDD based on age group (40 – 59 years vs. 60 or more years) and sex (male vs. female).**

**Figure S7. Spearman correlations between chronological age, proteomic and epigenetic aging clocks**

**Figure S8. Spearman correlations between proteomic and epigenetic aging clocks after adjusting for chronological age in linear regression models**

**Figure S9. Mendelian randomization analysis for the effects of PAC, HPS, and brain proteomic aging clock on MDD**

**Figure S1. Spearman correlations among systemic and organ-specific proteomic aging clocks after adjusting for chronological age in linear regression models.**


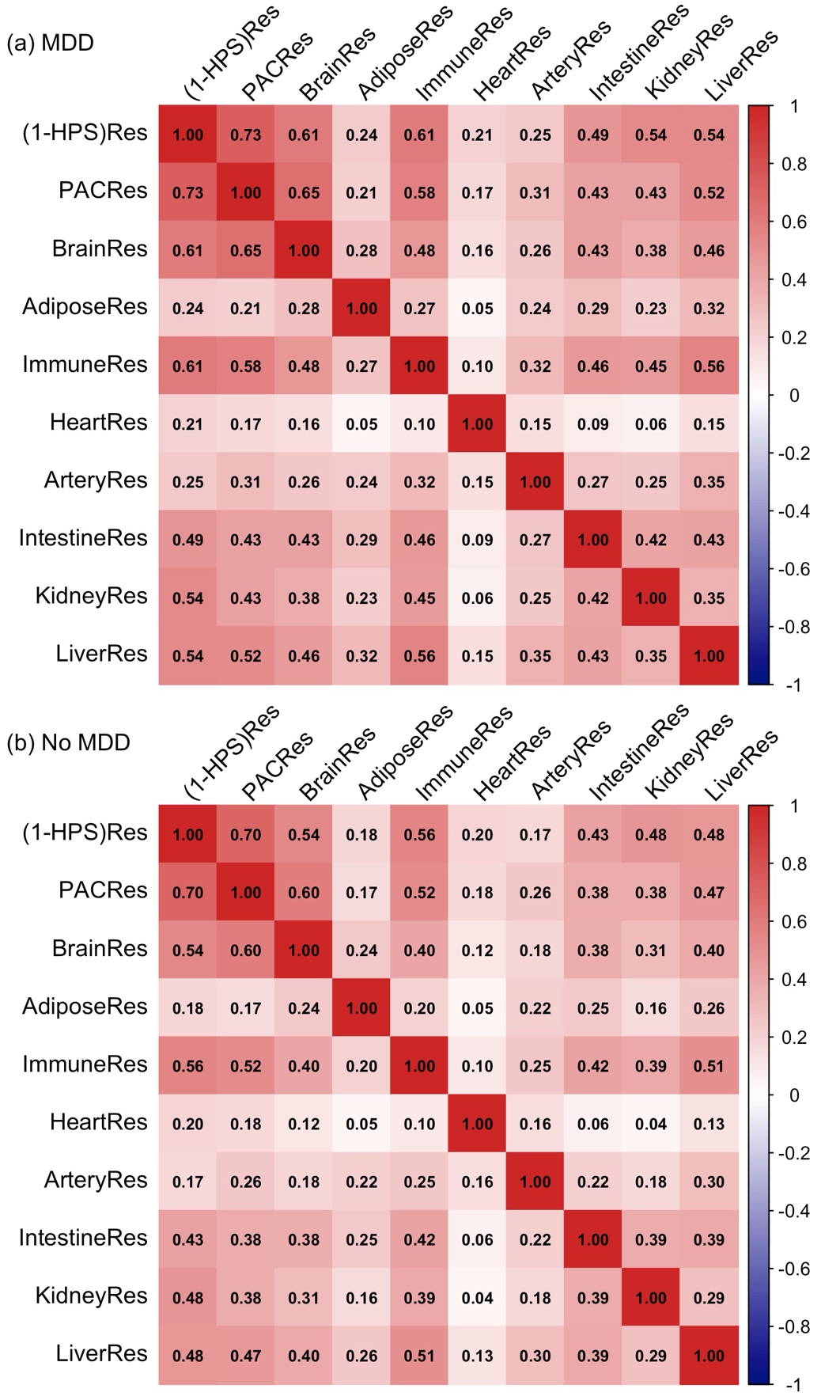


**Figure S2. Associations between proteomic aging (PA) measures (z-scores) and a history of MDD at baseline after adjusting for covariates (Active: diagnosed with MDD and PHQ-4 positive; Remitted: diagnosed with MDD and PHQ-4 negative; Never Dep: never diagnosed with MDD)**


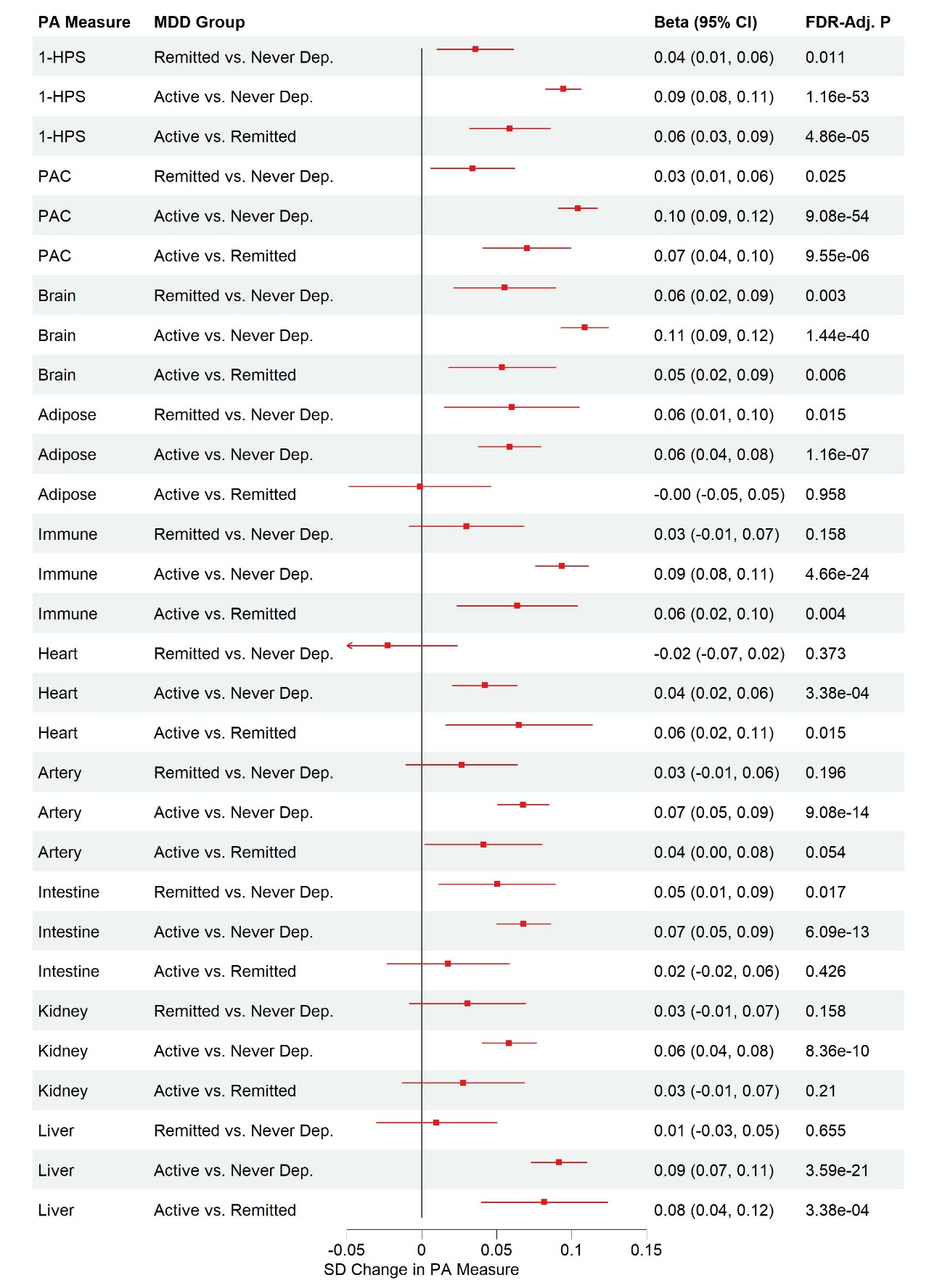


**Figure S3. A. Associations between antidepressant use (users vs. non-users) and proteomic aging (PA) measures (z-scores) in participants with MDD at baseline after adjusting for covariates (MDD Diag.: diagnosed with MDD before or at baseline; Active: diagnosed and PHQ-4 positive; Remitted: diagnosed and PHQ-4 negative). B. Association between specific antidepressant classes and proteomic aging (PA) measures (z-scores) in participants with MDD at baseline after adjusting for covariates. In both analyses, the reference group is Antidepressant use (No).**

1. **Prescribed antidepressants (Yes vs. No)**


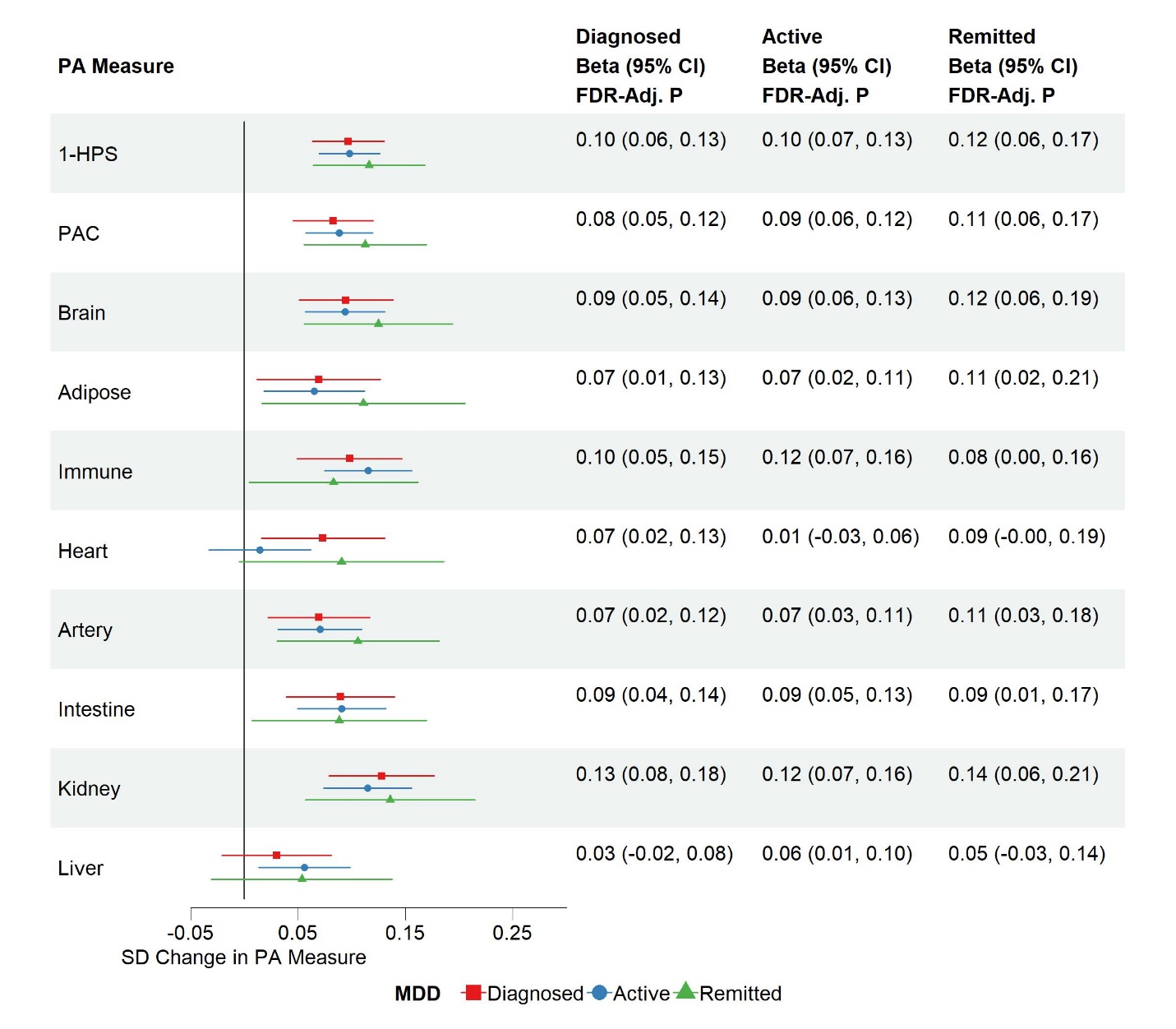


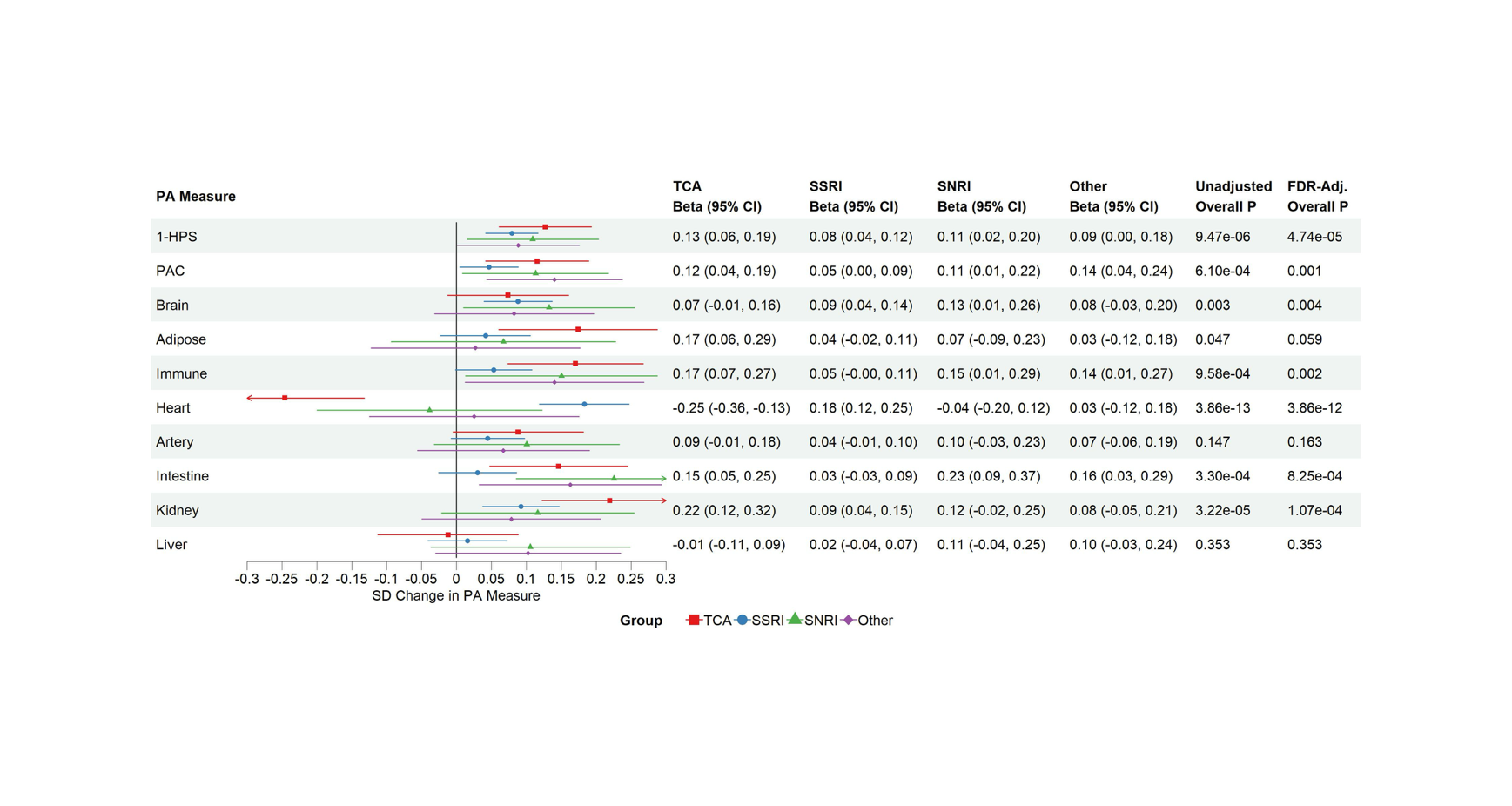
**B. Analyses based on major antidepressants classes prescribed.**

**TCA: tricyclic antidepressants; SSRI: selective serotonin reuptake inhibitors, SNRI: serotonin and noradrenalin reuptake inhibitors; Other antidepressants (including mirtazapine, bupropion).**

**Figure S4. Spearman correlations between the residuals of HPS, PAC, the brain proteomic aging clock, and cognitive function measures. Significance levels: p < 0.05 (*), p < 0.01 (**), p < 0.001 (***)**
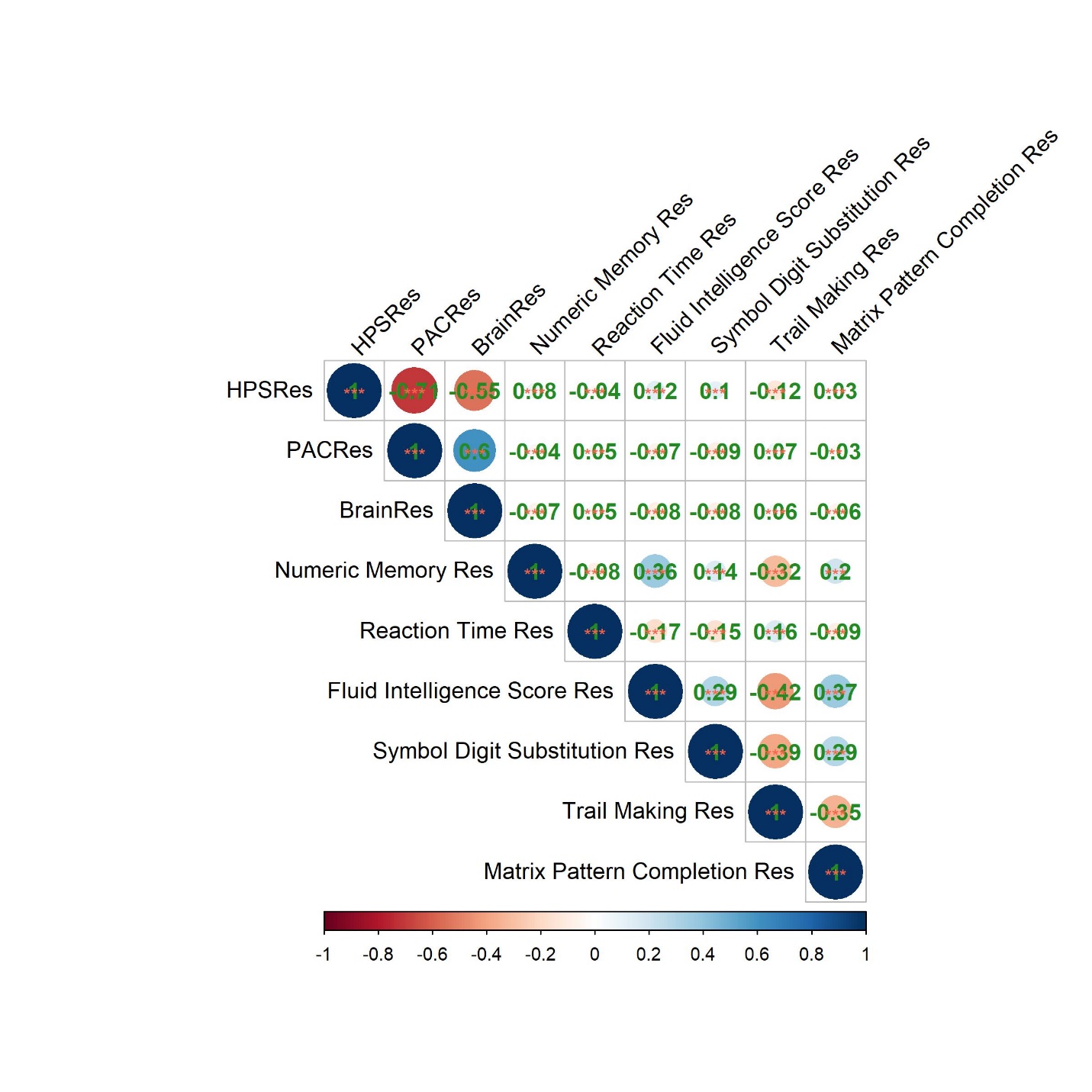


**Figure S5. Spearman correlations between the residuals of HPS, PAC, the brain proteomic aging clock, and brain MRI image-derived phenotypes (IDPs). IDPs are labeled if the FDR-adjusted p-value < 0.05 and the absolute Spearman correlation > 0.07, selected to ensure meaningful effect sizes while maintaining clarity**


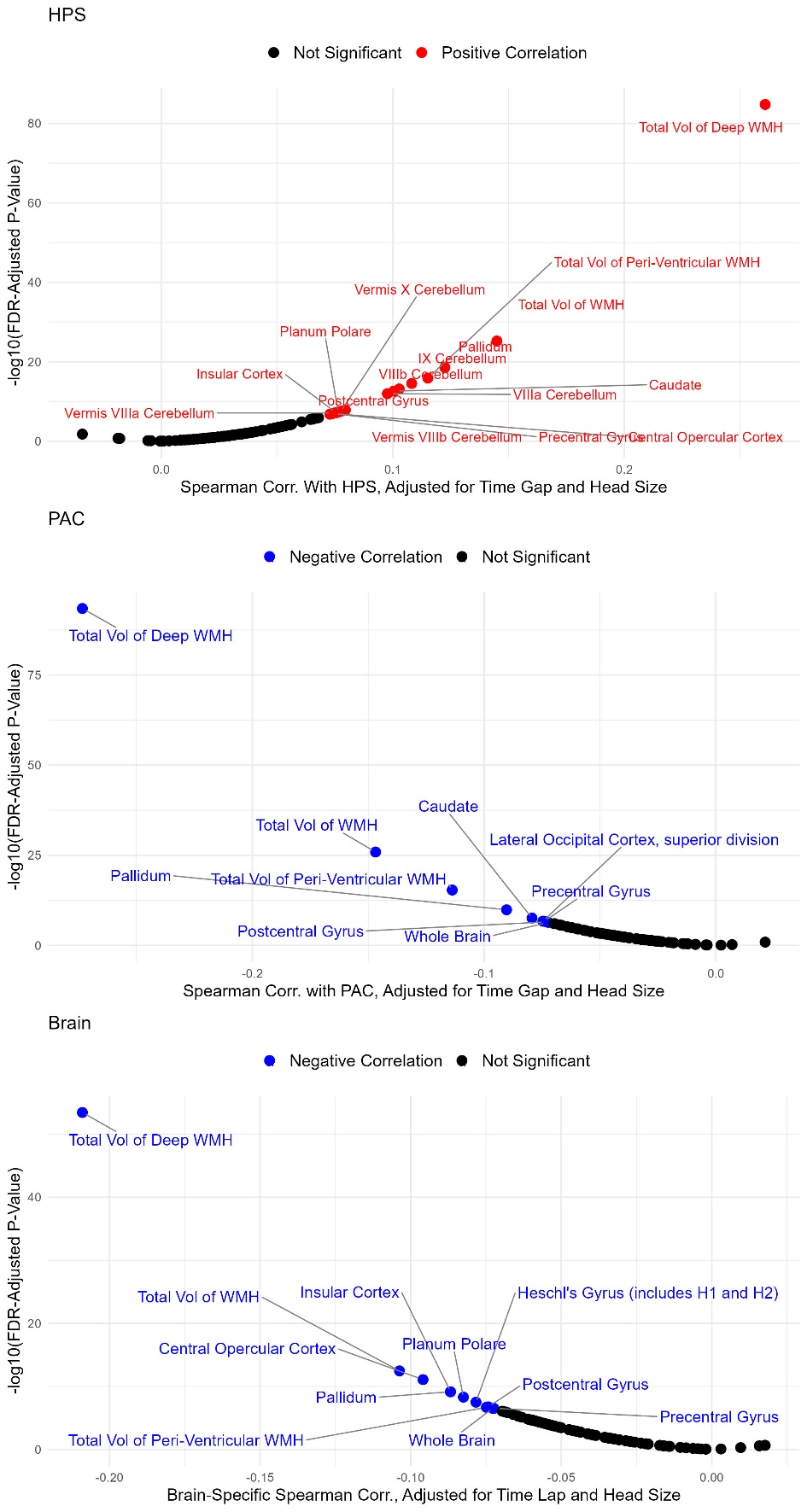


**Figure S6. Biological aging acceleration in MDD based on age group (40 – 59 years vs. 60 or more years) and sex (male vs. female).**

1.
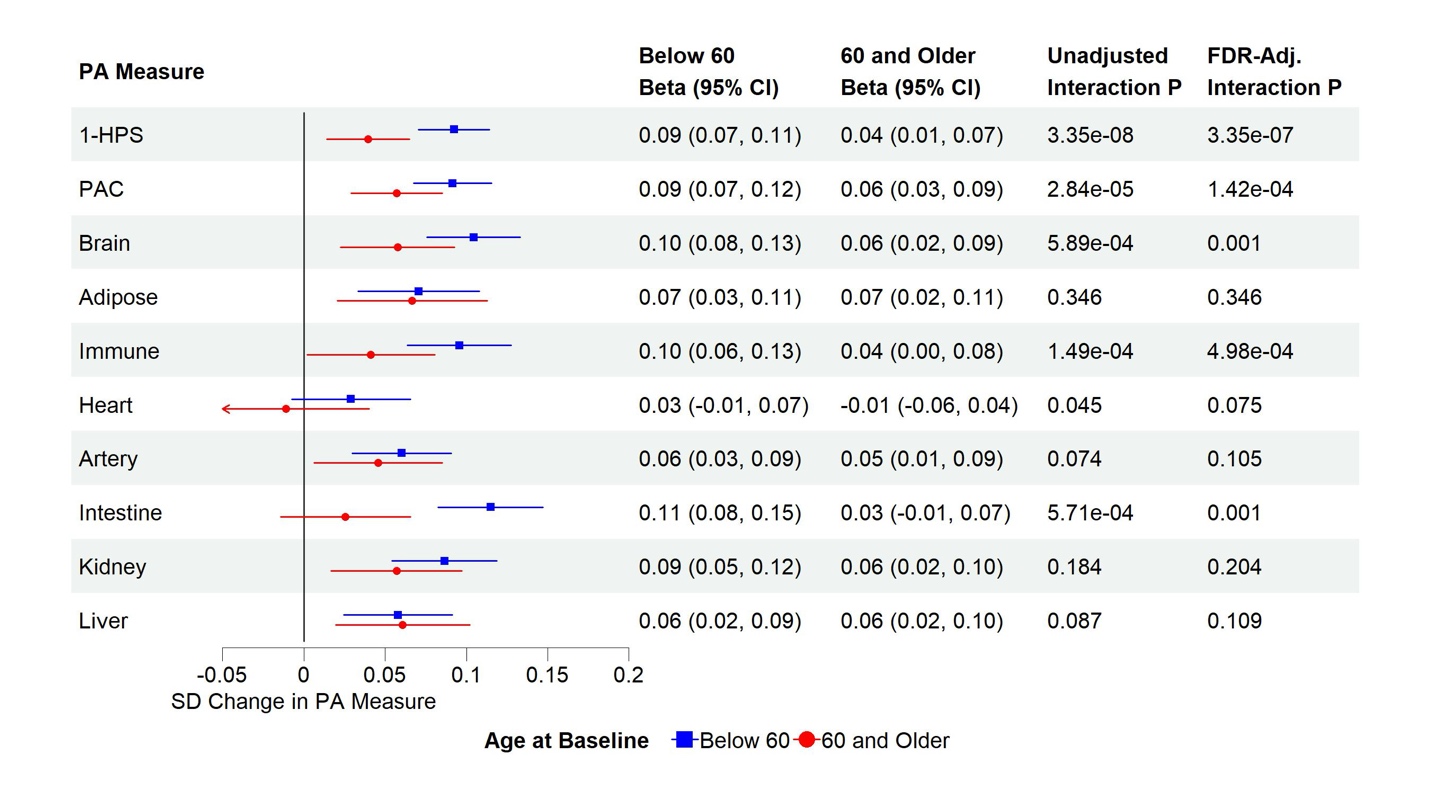
**Age group (40 – 59 years vs. 60 or more years)**
2. **
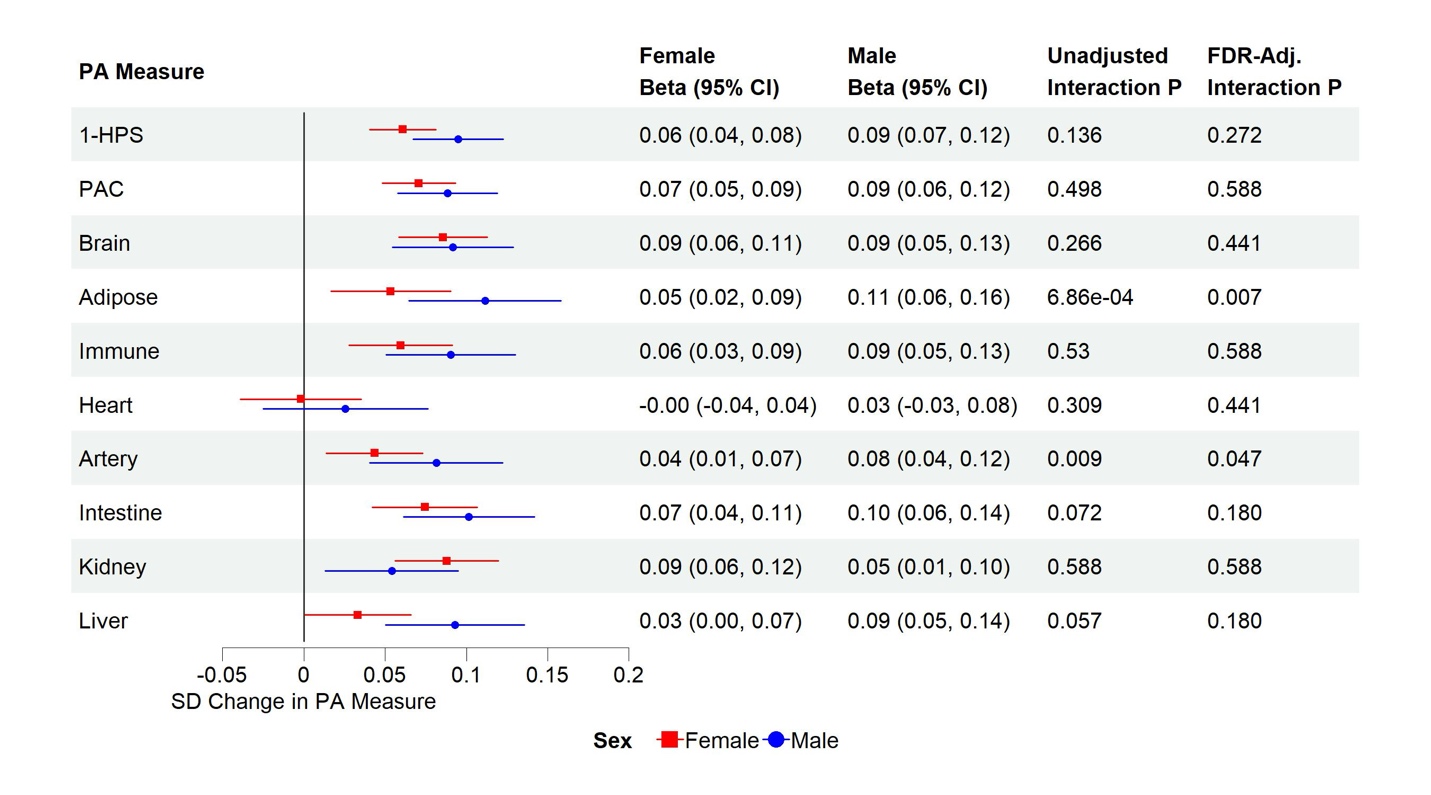
Sex (male vs. female)**

**Figure S7. Spearman correlations between chronological age, proteomic and epigenetic aging clocks**


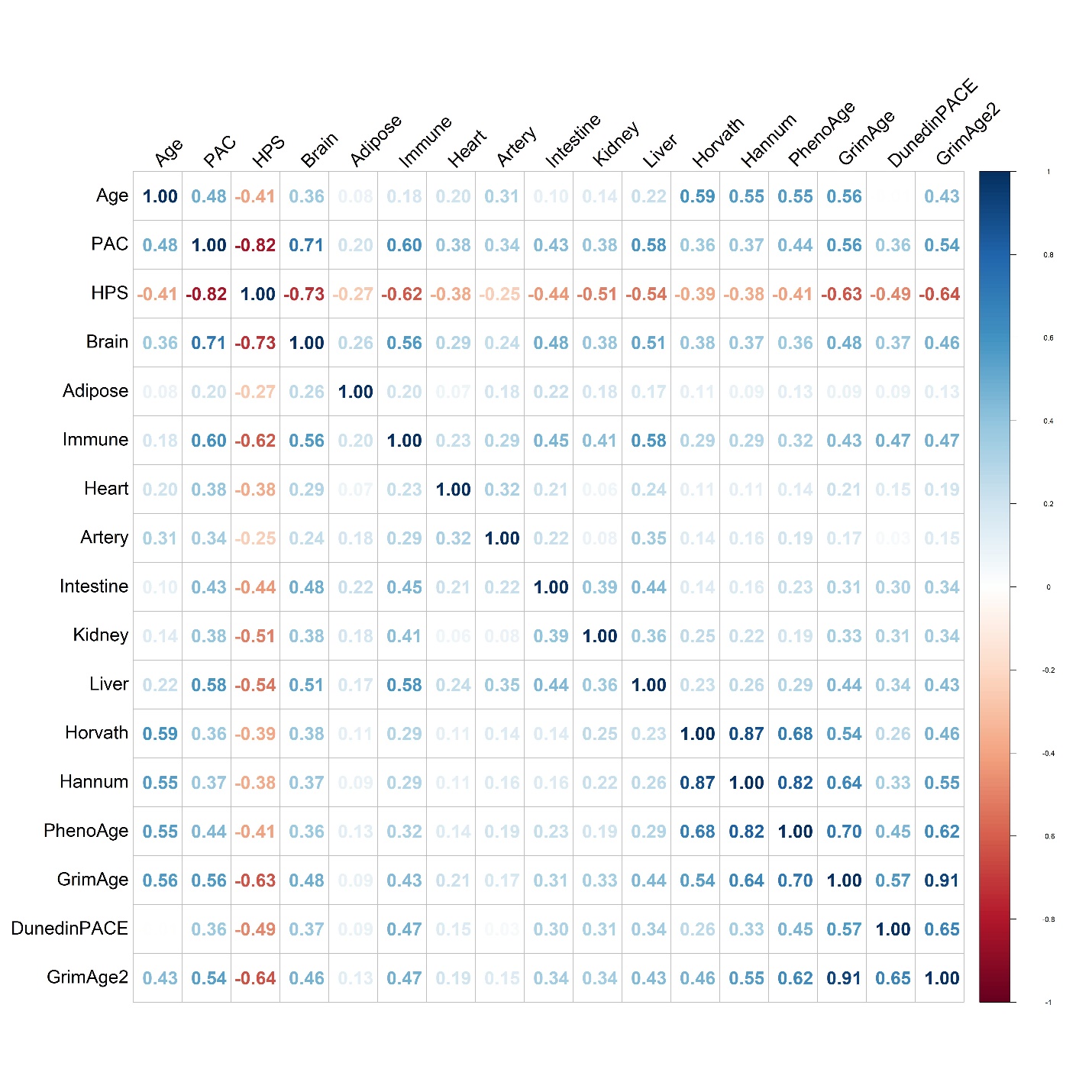


**Figure S8. Spearman correlations between proteomic and epigenetic aging clocks after adjusting for chronological age in linear regression models**


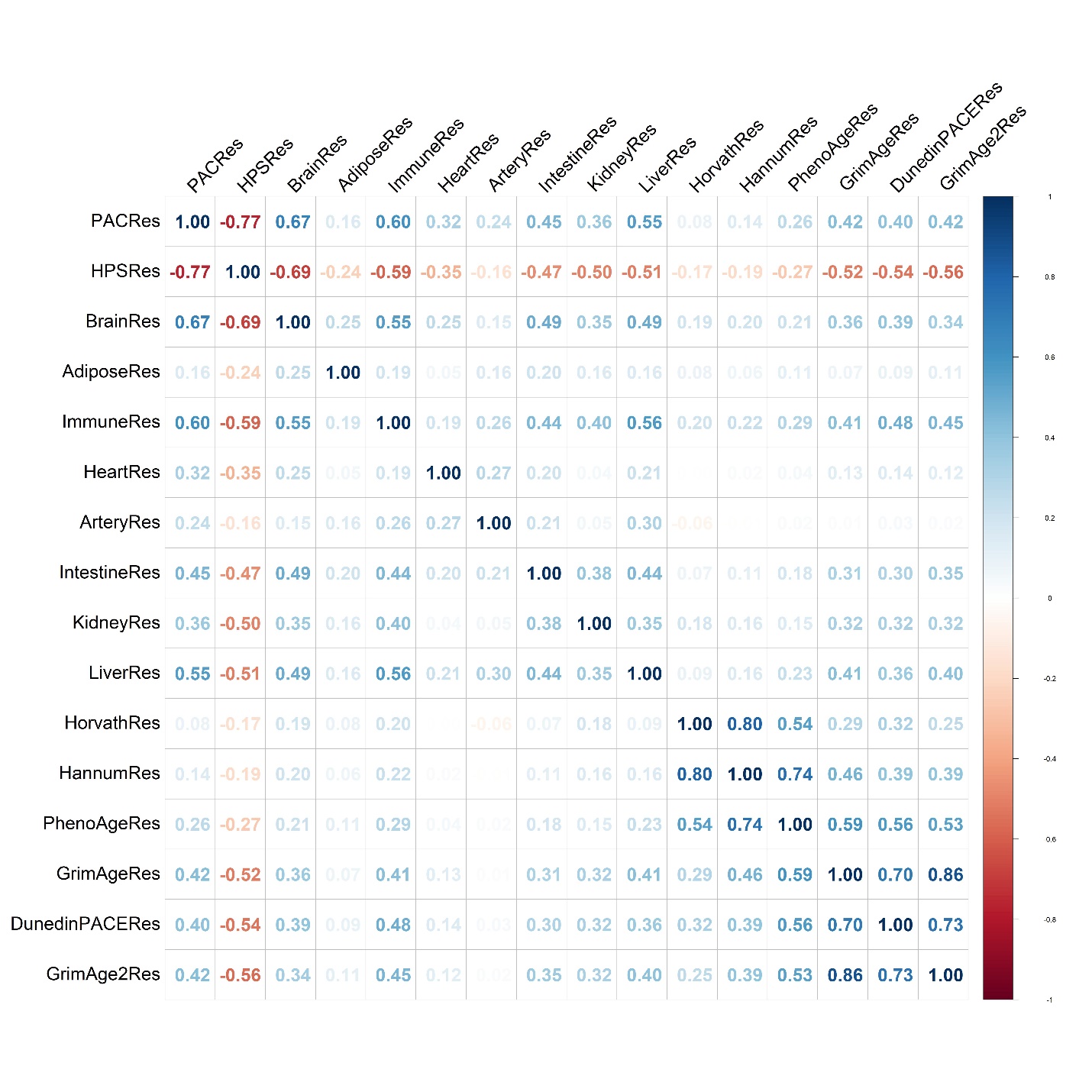


**Figure S9. Mendelian randomization analysis for the effects of PAC, HPS, and brain proteomic aging clock on MDD**

**
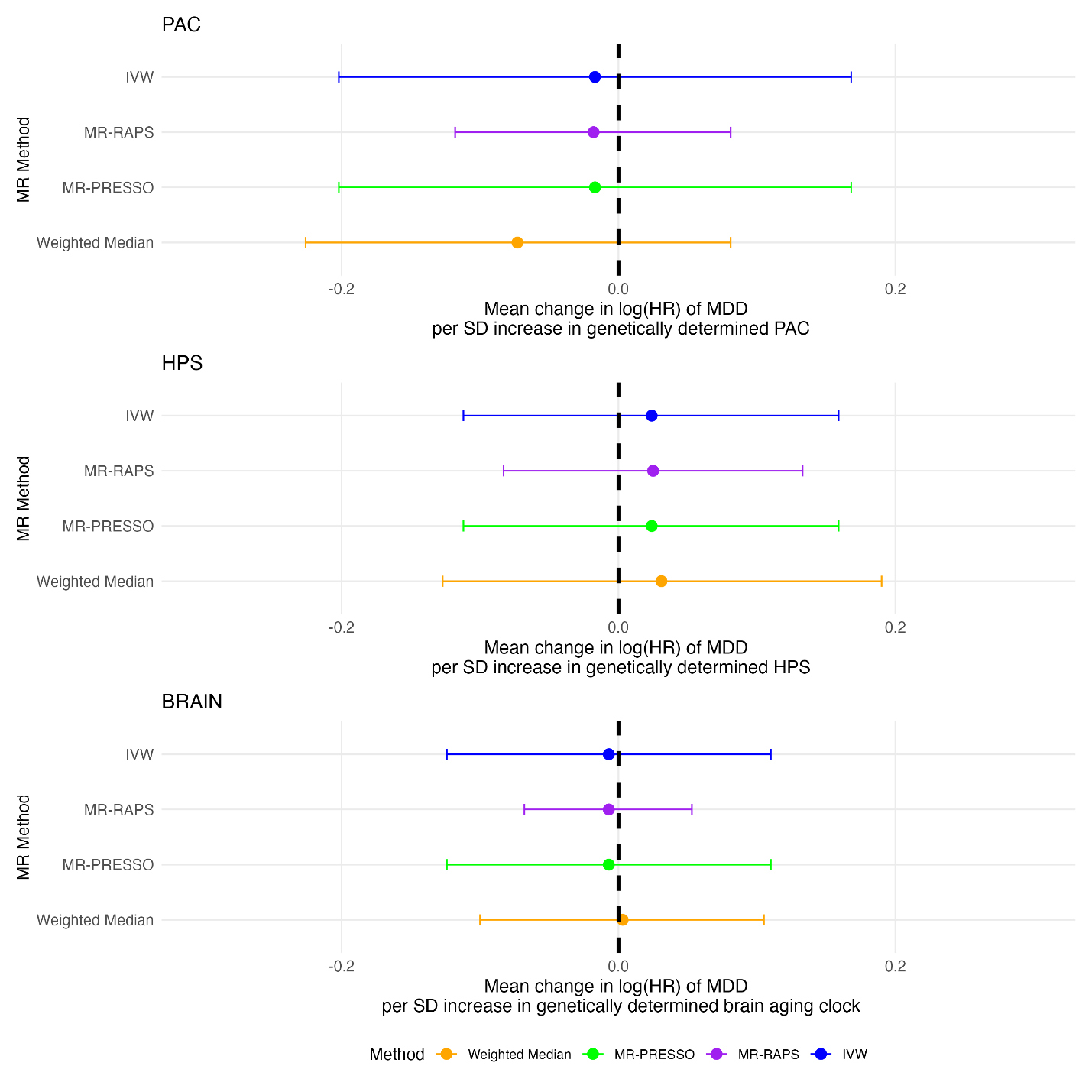
**
